# An Innovative Kefir Produced from Lactose Hydrolyzed Goat Milk with the Addition of the Probiotic *Saccharomyces Boulardii*: Volatile Compound Profile, Rheological and Sensory Properties

**DOI:** 10.1007/s12602-026-11002-0

**Published:** 2026-03-26

**Authors:** Ali Koçak, Tuba Şanlı, Canan Altınay, Ali Tekin, İsmayil Safa Gürcan, Ali Adnan Hayaloğlu

**Affiliations:** 1 Graduate School of Natural and Applied Science, Ankara University, Ankara, 06000 Turkey; 2 Department of Dairy Technology, Faculty of Agricultural, Ankara University, Ankara, 06000 Turkey; 3 Department of Food Technology, Vocational School of Keban, Fırat University, Elazığ, 23000 Turkey; 4 Department of Biostatistics, Faculty of Veterinary Medicine, Ankara University, Ankara, 06000 Turkey; 5 Department of Food Engineering, Faculty of Engineering, İnönü University, Malatya, 44280 Turkey

**Keywords:** Fermented milk, Small ruminant milk, Lactose hydrolysis, Organic acids in fermented dairy products, *Saccharomyces boulardii*

## Abstract

The rationale for this study was that using *Saccharomyces boulardii* as a probiotic, particularly in kefir and similar fermented milk products made from goat’s milk, could provide an innovative approach to enhancing health benefits and overall sensory acceptability. This study investigates the incorporation of *S. boulardii* into lactose-hydrolyzed goat’s milk for kefir production and evaluates its impact on functional, microbiological and sensory characteristics during 28-day storage period. Goat’s milk to be used in the production of lactose-reduced and lactose-free alternatives was subjected to 50% and 100% lactose hydrolysis with β-galactosidase, and kefir samples were produced with and without the addition of *S. boulardii*. The results showed that, increased glucose and galactose content due to lactose hydrolysis improved fermentation efficiency. *Lactobacillus* spp. counts peaked at 8.57 log CFU/mL in LH100B, while *S. boulardii* maintained viable levels above 10⁶ CFU/mL throughout storage, meeting probiotic criteria. Proteolytic activity and antioxidant capacity increased significantly, especially in lactose-hydrolyzed samples with *S. boulardii*. Antioxidant capacity (ABTS assay) reached its highest value on day 14, showing up to 28–30% higher radical scavenging activity in S. *boulardii* containing samples compared to control. The level of lactose hydrolysis, particularly in combination with *S. boulardii*, significantly influenced technological and biochemical properties. The highest water-holding capacity was observed in the 100% hydrolyzed sample with *S. boulardii*, while lactose hydrolysis increased the consistency index without affecting flow behavior. Although hydrolysis alone did not markedly alter the volatile profile, samples containing *S. boulardii* were characterized by alcohol-dominant volatiles. Additionally, lactose hydrolysis reduced citric and malic acids, whereas acetic acid levels remained elevated in samples containing *S. boulardii* throughout storage. Sensory evaluation showed that kefir produced from 50% lactose-hydrolyzed goat’s milk with *S. boulardii* received the highest overall acceptability score from the panelists, while samples with 100% lactose hydrolysis also maintained favorable sensory characteristics. The results suggest that the combination of lactose hydrolysis and incorporation of *S. boulardii* enhances the functional, rheological, and sensory properties of kefir made from goat’s milk, offering a promising strategy for developing fermented dairy products suitable for lactose-intolerant consumers and the functional food market.

**Supplementary Information:**

The online version contains supplementary material available at 10.1007/s12602-026-11002-0.

## Introduction

Kefir is a functional fermented milk product that has been consumed for thousands of years and contributes significantly to human health [1]. It is produced through the symbiotic fermentation of lactose-fermenting and non-lactose-fermenting yeasts with lactic and acetic acid bacteria [2, 3]. Kefir is traditionally produced using kefir grains, and commercially using pure cultures [4]. While primarily made from cow’s milk, kefir produced from sheep [5], goat [5, 6] and buffalo [7] milk has also been studied. It has also been reported that kefir is produced using non-animal milks such as soy milk and coconut milk [8].Variations in milk composition influence the quality and health benefits of kefir [9]. Goat’s milk, in particular, is a nutritionally rich and health-promoting alternative to conventional cow’s milk. Goat’s milk has distinct compositional and physicochemical properties that make it suitable for use in fermented milk products such as kefir. Specifically, various carbohydrates in goat’s milk such as galactosyllactose and sialyloligosaccharides have prebiotic properties that support the growth of beneficial gut bacteria and can alleviate symptoms of digestive disorders [8, 10]. It has been identified as an effective carrier for maintaining probiotic viability during shelf life [11]. Probiotic microorganisms are expected to remain viable at 10⁶–10⁹ CFU/g or CFU/mL during processing, storage, and digestion [12, 13], and the International Dairy Federation (IDF) recommends at least 10⁷ CFU per gram at consumption. Moreover, probiotics may improve the sensory attributes of goat’s milk by reducing the “goaty” flavor associated with short-chain fatty acids [14–16].

Probiotic microorganisms can include bacteria, molds, or yeasts, although bacteria are the most commonly utilized [17]. Beneficial probiotic bacteria primarily belong to the genera *Lactobacillus*, *Bifidobacterium*, *Enterococcus*, *Streptococcus*, *Pediococcus*, *Leuconostoc*, *Bacillus*, and *Escherichia*, while *Saccharomyces* is the only yeast genus with confirmed probiotic properties [18]. Among them, *S. boulardii* has been clinically validated, classified as safe by the European Food Safety Authority (EFSA), and is the only probiotic yeast approved by the US FDA for human consumption [16, 19]. Unlike probiotic bacteria, *S. boulardii* is not part of the gut flora. However, it resistant to gastric acid, bile salts, and pancreatic fluids. This enables *S. boulardii* to remain viable at low pH values ​​and withstand antibiotic treatments [14, 15, 18, 17]. Clinical studies have shown that *S. boulardii* provides many health benefits, including producing antibacterial molecules, supporting the growth of probiotics, generating immune-boosting substances, stimulating the development of gut-related cells, increasing the production of digestive enzymes such as sucrase, lactase, and maltase, enhancing short-chain fatty acid production, and preventing inflammation in the body [19, 20]. These features position *S. boulardii* as a promising candidate for the development of novel functional products.

Since *S. boulardii* cannot ferment lactose, it is employed as a secondary culture in fermented products, initiating growth after lactic acid bacteria hydrolyze lactose into glucose and galactose [21, 22]. The high concentration of galactose in the environment creates an optimal growth medium for *S. boulardii*, which is lactose-negative but galactose-positive [15, 23]. *S. boulardii* exhibits greater resistance to low pH than other Saccharomyces strains, enhancing its stability during storage; additionally, it promotes the growth and viability of lactic acid bacteria throughout the storage period [21, 22]. This increased activity in fermented products has been reported to contribute to the development of functional properties [23, 24].

Lactose-hydrolyzed dairy products are produced by breaking down lactose, a disaccharide in milk, into glucose and galactose using the enzyme lactase (β-galactosidase). This process can alter both sensory and technological properties [25, 26]. Lactose-free dairy products provide an alternative for individuals with lactose intolerance and enable the development of new flavors.

While there are several studies on *S. boulardii* in dairy products, research on its use in fermented dairy products is limited. Most studies focus on yogurt [14–16, 27], fermented milk [21], and cheese and whey [28–31]. Only a few studies have examined the addition of *S. boulardii* to kefir production [32, 33], and, as in the present study, there is no detailed study examining the physicochemical, biochemical, and microbiological properties of kefir samples produced from lactose-hydrolyzed goat milk using *S. boulardii* as the sole probiotic yeast. To address this gap in the literature and provide new data in this field, the present study also aimed to: investigate possible changes in the antioxidant activity of kefir during storage depending on the addition of *S. boulardii*; and produce a consumer-favored kefir by integrating lactose hydrolysis and *S. boulardii* into goat milk used as the raw material in kefir production. In this context, the taste and aroma of kefir, as well as the volatile aroma components and organic acids affecting them, were also analyzed.

## Materials and Methods

### Materials

The raw goat milk used for the production of kefir was obtained from Mehmet Bey Organic Goat Farm (Bayramgazi Village, Afyonkarahisar, Turkey). Kefir production took place in the research laboratory of the Dairy Technology Department of the Faculty of Agriculture at Ankara University. The starter cultures, DVS Hansen eXact Kefir-2 and XPL-1 mesophilic/thermophilic mixed cultures, which were purchased from Chr. Hansen, were used. For the *S. boulardii* strain, the freeze-dried powder culture CNCM I-745 (Reflor, Biocodex-France) was used. A microbial ß-galactosidase enzyme (NOLA^TM^ Fit, Chr. Hansen, 5500\5 L) was used for lactose hydrolysis.

### Lactose Hydrolysis and Kefir Production

Prior to the main study, preliminary trials were conducted to evaluate the effect of different lactose hydrolysis levels (25%, 50%, 75%, and 100%) on the quality characteristics of goat milk kefir. Based on the preliminary results regarding water-binding capacity, viscosity values, and sensory evaluation, a 50% hydrolysis rate was selected for the reduced-lactose product and a 100% hydrolysis rate for the lactose-free product. Accordingly, lactose hydrolysis in the main study was performed at these two levels (50% and 100%) using β-galactosidase, based on supplier recommendations (LactoSens, DirectSens GmbH, Austria). Milk was incubated at 10 °C for 12 h for lactose hydrolysis, followed by enzyme inactivation at 95 °C for 5 min. The degrees of hydrolysis achieved were confirmed using the LactoSens test kit.

**Fig. 1:**
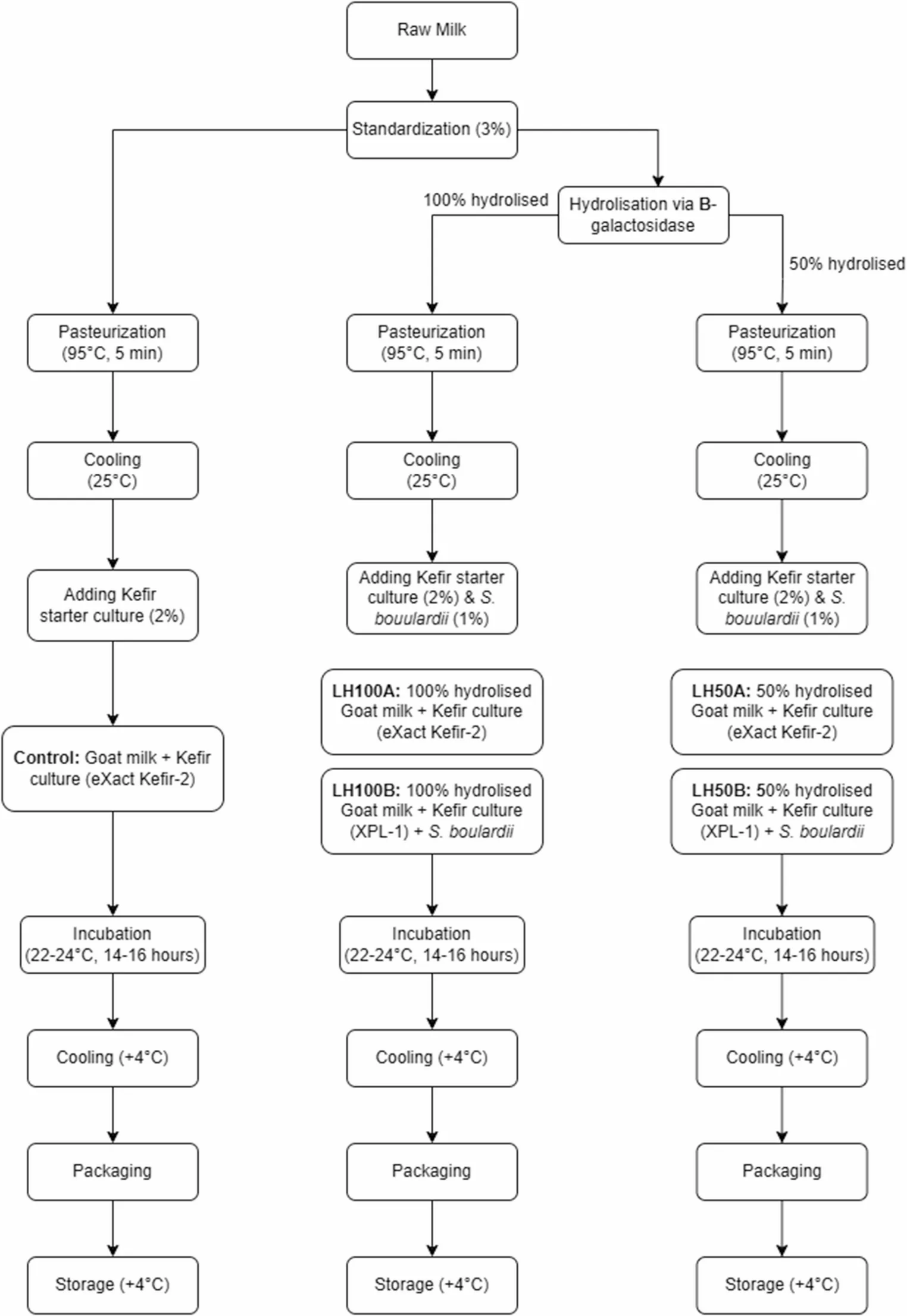
Flowchart of the kefir production process

Raw goat’s milk (total solids 10.9%, fat 3.2% and protein 4.21%) was divided into three parts, one part being reserved for the preparation of the control sample. The second and third parts were subjected to 100% and 50% lactose hydrolysis, respectively. After this step, the milk portions were heat-treated at 90 °C for 5 min and then cooled to 25 °C. The second and third portions were then aseptically divided into two parts, one part was inoculated with *S. boulardii* and kefir starter culture and the other part received only the kefir starter culture, as was done with the milk for the control sample. All samples were then incubated at 22 °C for about 16 h until the pH value reached 4.6. Five different kefir samples were obtained in this way, coded as follows: C (control kefir), made from milk without added ß-galactosidase; LH100A and LH100B, kefir samples made from milk with 100% lactose hydrolysis, without and with *S. boulardii*, respectively; LH50A and LH50B, kefir samples made from milk with 50% lactose hydrolysis, without and with *S. boulardii*, respectively. The kefir samples were filled into 1-liter glass bottles and stored at + 4 °C for 28 days. The physicochemical, rheological, microbiological and sensory properties of the kefir samples were analyzed on the 1st, 7th, 14th, 21st and 28th day. The total solids, protein and fat content of the kefir samples ranged from 10.84 to 11.04%, 4.21–4.25% and 3.20–3.25%, respectively. Figure 1 shows the flow chart of the production process of the kefir samples.

### Chemical Composition and pH

The dry matter (gravimetric), fat and protein content of the kefir samples was determined according to Hooi et al. [34] The pH values were measured by digital calibrated pH meter (Ohaus, Starter 300, USA).

### Proteolytic Activity and Antioxidant Capacity

The method described by Azizkhani et al. [2] was used to separate water-soluble nitrogenous substances used in the determination of total free amino acid content and antioxidant activity of kefir samples.The total free amino acid content in the kefir samples was determined using the ortho-phthalaldehyde (OPA) method as reported by Ayyash et al. [35]. The absorbance measurements were performed at a wavelength of 340 nm using a UV-VIS spectrophotometer (PerkinElmer, Lambda 25, Singapore), and the results were expressed as absorbance (Å) values.

The antioxidant activity of peptides produced by the in vitro hydrolysis of proteins is related to the release of more ionizable groups and hydrophobic groups. It has been reported that peptides in protein hydrolysates act as antioxidants through free radical scavenging mechanisms, inhibition of lipid peroxidation, chelation of metal ions or a combination of these mechanisms and that antioxidant activity should be evaluated by different methods [36]. Therefore, three different methods were used to determine the antioxidant activity of the samples: ABTS (2,2’-azino-bis(3-ethylbenzothiazoline-6-sulfonic acid)), DPPH (2,2-diphenyl-1-picrylhydrazyl) and FRAP (Ferric Reducing Antioxidant Power).

The ABTS assay was performed according to the method outlined by Köse and Ocak [37], and the results were expressed as Trolox equivalents (µmol Trolox/g sample). The DPPH assay was conducted following the protocol of Apostolidis et al. [38], with absorbance measured at 517 nm. FRAP (Ferric Reducing Antioxidant Power) method was applied as described by Çelik et al. [39], and the results were expressed as Trolox equivalents (µmol Trolox/g sample).

### Carbohydrate and Organic Acid Contents

The content of carbohydrates and organic acids was determined simultaneously by HPLC (LC Prominence model; Shimadzu, Kyoto, Japan) using a photodiode array detector (at 210 nm) and a refractive index detector [40]. For analysis, 1 g of each kefir sample was diluted with 5 mL of 45 mM H_2_SO_4_ and centrifuged at 5500 g for 20 min at 4 °C (Hettich 320 R, Tuttlingen, Germany). The supernatant was then filtered through a filter (Syringe Filter Nylon, 25 mm x 0.45 μm filter, Millipore) and transferred to a vial; 20 µL was used for analysis.

### Rheological Properties

Rheological tests are crucial for investigating liquid flow behavior and characterizing product structure by determining shear rate and stress, enabling the creation of a flow model [41]. In this study, rheological analysis of kefir samples was performed using a Kinexus Pro+ rheometer (Malvern, Worcestershire, UK). Kefir samples were mixed at a constant speed for 30 s at 4 °C, and viscosity was measured using a 4° conical probe (20 mm diameter, 1 mm gap) across a shear rate range of 0.10–300 s⁻¹ at 4 °C. The data were analyzed to calculate the flow index (n) and consistency index (K).

The shear rate-shear stress graph indicated that kefir exhibited non-Newtonian flow behavior. Flow models, including the Newtonian and Power-Law models, were tested, with the Power-Law model providing the best fit (R² > 0.99). The Power-Law equation used for calculations is

$$\:{\upsigma\:}=K{{\upgamma\:}}^{n}$$

Where 𝜎 represents shear stress, K is the consistency index, 𝛾 is the shear rate, and n is the flow behavior index.

### Water Holding Capacity (WHC)

The WHC in kefir samples was determined according to the principle of phase separation using the method described by Isanga and Zhang [42]. 25 g of kefir samples were weighed into a test tube and centrifuged at 8000 g for 15 min at 5 °C (Sigma, model 3–18 K, Germany). The WHC was expressed using the following formula.

$$\:\mathrm{W}\mathrm{H}\mathrm{C}\:\left(\mathrm{\%}\right)=\left(1-\left({W}_{1}-\:{W}_{2}\right)\right)*100$$

Where, W_1_=weight of the whey after centrifugation and W_2_=kefir initial weight.

### Color

Color The L*, a* and b* values, which express the color properties of the kefir samples, were measured with a chroma meter colorimeter (Konica Minolta CR 410, Sensing Inc., Osaka, Japan). According to the Hunter colorimetric system, the L* value, as a color parameter symbolizing lightness, varies between 100 and 0 and expresses the transition from lightness to darkness. Among other color properties, the a* value is considered an indicator of red (+) or green (-), and the b* value is considered an indicator of yellow (+) or blue (-) [43].

### Volatile Compounds

The volatile components in the kefir samples were determined by solid phase microextraction (SPME) method. 5 g of the kefir sample was weighed into a 40 mL SPME vial (Supelco, USA) and 10 µL of internal standard (100 ppm 2-methyl-3-heptanone and 300 ppm 2-methyl-1-pentanoic acid in methanol) and 0.5 g NaCl were added. The samples were stored at -18 °C until analysis. Prior to injection, the vials were stored at 40 °C for 30 min on a heater (PC-420D, Corning, NY, USA) to allow the volatiles to be absorbed onto the SPME fiber (50/30 µm DVB/carboxes/PDMS, Supelco, Bellefonte, PA) [44]. The separation of volatile components adsorbed on the fiber was performed using a gas chromatography-mass spectrometer (GC-2010/MS-QP-2010, Shimadzu Corp., Kyoto, Japan). The volatiles were separated using a DB-Wax column (60 m × 0.25 mm × 0.25 μm), operated in splitless mode, and mass spectra were scanned in the range of 33–450 eV. The column temperature program for chromatography was applied as follows: initial 40 °C for 3 min, heating to 70 °C with 5 °C/min increments and holding for 1 min, heating to 240 °C with 10 °C/min increments and holding for 5 min. The mass spectra of the volatile components were identified by comparison with data from Wiley 9 (9th Edition, John Wiley & Sons, Inc., 2005) and NIST14 (NIST/EPA/NIH, 2014) mass spectral laboratories. In addition, reference standards (Sigma Aldrich, St. Louis, MO) for acetaldehyde, ethanol, diacetyl, acetoin and butanoic acid were injected into the chromatographic system under the same conditions. The retention indices (RI) of each compound were calculated using a series of n-alkanes (C10–C26) under the same chromatographic conditions to confirm their identity. The calculated RI values were compared with literature data [44]. The concentration of each volatile component was expressed in mg/L.

### Microbiological Analyses

Sabouraud dextrose agar (SDA) was used to determine the number of *S. boulardii*, M17 agar was used to determine the number of *Lactococcus* spp. petri dishes were incubated at 37 °C for 48 h. The number of *Lactobacillus* spp. was determined using De Man Rogosa Sharpe Agar (MRS) after 72 h of anaerobic incubation at 37 °C. The results were expressed as log colony forming units per gram of sample (log CFU/mL).

### Sensory Evaluation

The sensory panel consisted of 7 semi-trained panellists composed of scientists from the Department of Dairy Technology, Faculty of Agriculture, Ankara University, Ankara, Turkey. All samples were presented with a three-digit code. *A multiple comparison test was conducted to evaluate the sensory quality (taste*,* texture*,* and overall acceptability) of the kefir samples using a scoring scale ranging from 1 “extremely dislike” to 9 “extremely like”* [45]. The study was conducted in accordance with the World Medical Association’s Code of Ethics (Declaration of Helsinki). According to the Institutional (Ankara University, Turkey) rules, ethical approval was not required for the sensory analysis, as all kefir samples used were safe and commonly consumed. Participation in the sensory evaluation was entirely voluntary. Panel members were informed that they would not receive financial compensation for their involvement and that they could withdraw from the study at any point without providing a reason. It was also emphasized that all data and individual performance results would remain confidential, and no names or personally identifiable information would be disclosed or published.

### Statistical Analysis

When evaluating the data, the statistical analysis was carried out with the GLM method of two-way analysis of variance (two-way ANOVA) using the SPSS 14.01 package program (license number: 9869264). Kefir-making process and each experiment were repeated in twice. Statistically significant differences were determined at the *P <* 0.05 level using the Duncan multiple comparison test.

## Result and Discussion

### Chemical Composition, pH and Proteolytic Activity

The dry matter (10.84–11.04%), protein (4.21–4.25%), and fat (3.20–3.25%) contents showed minimal variation among treatments, indicating that lactose hydrolysis and *S. boulardii* addition did not markedly alter the basic chemical composition of kefir. The chemical composition of the kefir samples was comparable to the values reported by Saygılı et al. [5] and Gürel et al. [6] for kefir produced from goat’s milk. In dairy products, the pH, which is a fundamental factor for the fermentation activity of the starter culture and plays an important role in the quality characteristics of the product, varies with the incubation conditions such as temperature and time, the growth rate and fermentation capacity of the microorganisms of the starter culture and the composition of the milk [46]. While the pH values varied between 4.47 and 4.53 on the first day of storage, they steadily decreased with increasing storage time (*P <* 0.05) and were between 4.35 and 4.45 on the 28th day of storage (Table 1). The more pronounced decrease observed in LH100A and LH50A samples suggests that lactose hydrolysis accelerated acid production, likely due to the rapid utilization of released glucose by lactic acid bacteria. It is considered that the results obtained are consistent with those of some studies [41, 47, 48]; however, different pH values may be obtained depending on factors such as the microorganisms used in the starter culture and storage conditions. Similarly, Baniasadi et al. [46] reported that the pH changes observed during the storage time of kefir and yogurt samples are due to the buffering capacities of the milk used and the differences in the fermentation capacity of the different microbial populations. The lowest pH values during storage were found in kefir samples (LH100A and LH50A) produced from 100% to 50% lactose hydrolyzed milk. This result can be explained by the fact that glucose, which is a free energy source due to lactose hydrolysis, is more easily metabolized by the bacteria in the starter culture and the acidity level increases faster [49].

**Table 1:** pH values, total free amino acid contents, antioxidant capacity (ABTS assay, DDPH assay and FRAP assay) and color properties of kefir samples during storage

Variables	Kefir samples						ANNOVA		
	Storage (Day)	C (Control)	LH100A	LH100B	LH50A	LH50B	*P*-sample	*P*-storage	*P*-sample ⋅ storage
pH	1	4.52 ± 0.01^aA^	4.50 ± 0.04^aAB^	4.53 ± 0.01^aA^	4.47 ± 0.01^aB^	4.52 ± 0.01^aA^	**	**	*
	7	4.47 ± 0.04^bA^	4.37 ± 0.02^bB^	4.43 ± 0.04 ^bAB^	4.40 ± 0.01^bB^	4.47 ± 0.01^bA^			
	14	4.41 ± 0.02^b^	4.35 ± 0.02^b^	4.39 ± 0.01^b^	4.39 ± 0.02^b^	4.40 ± 0.01^ba^			
	21	4.45 ± 0.02^bA^	4.31 ± 0.01^bC^	4.39 ± 0.03^bAB^	4.38 ± 0.02^bB^	4.44 ± 0.02^bA^			
	28	4.45 ± 0.02^bA^	4.36 ± 0.01^bD^	4.38 ± 0.01^bC^	4.35 ± 0.02^bD^	4.41 ± 0.01^baB^			
OPA values (Å)	1	0.28 ± 0.01^cB^	0.34 ± 0.03^bAB^	0.35 ± 0.02^bAB^	0.37 ± 0.01^bA^	0.37 ± 0.01^bA^	**	**	ND
	7	0.36 ± 0.01^b^	0.36 ± 0.02^b^	0.36 ± 0.03^b^	0.38 ± 0.01^b^	0.38 ± 0.01^ab^			
	14	0.37 ± 0.01^ab^	0.39 ± 0.01^ab^	0.39 ± 0.01^ab^	0.39 ± 0.01^ab^	0.39 ± 0.01^ab^			
	21	0.38 ± 0.01^abB^	0.41 ± 0.01^aA^	0.42 ± 0.02^aA^	0.41 ± 0.01^aA^	0.42 ± 0.02^aA^			
	28	0.39 ± 0.01^aB^	0.43 ± 0.01^aA^	0.43 ± 0.01^aA^	0.43 ± 0.01^aA^	0.43 ± 0.04^aA^			
ABTS assay (µmol Trolox/g sample)	1	109.88 ± 1.53^cB^	91.84 ± 5.20^cCD^	113.49 ± 2.64^cA^	95.44 ± 0.33^cC^	91.48 ± 1.48^dD^			
	7	81.01 ± 2.11^dB^	86.05 ± 0.46^dA^	88.23 ± 4.20^dA^	89.49 ± 3.46^A^	79.05 ± 2.27^eB^			
	14	138.74 ± 3.51^aC^	160.41 ± 3.27^aA^	142.36 ± 1.21^aC^	151.53 ± 0.30^aB^	147.93 ± 1.72^aC^			
	21	120.71 ± 1.98^bB^	135.20 ± 1.07^bAB^	127.73 ± 2.03^bA^	113.49 ± 0.61^bAB^	117.90 ± 2.60^bB^			
	28	84.62 ± 4.34^dC^	93.35 ± 1.76^cB^	95.27 ± 2.35^dB^	95.44 ± 1.39^cB^	104.58 ± 2.16^cA^			
DPPH assay (%)	1	61.50 ± 0.70	61.50 ± 0.70	61.00 ± 0.01	61.50 ± 0.70	60.50 ± 0.70	ND	ND	ND
	7	61.50 ± 0.70	61.00 ± 0.00	61.00 ± 0.01	60.00 ± 1.41	61.50 ± 2.12			
	14	60.00 ± 1.41	61.00 ± 1.41	61.00 ± 1.41	61.50 ± 0.70	61.00 ± 2.82			
	21	60.50 ± 0.70	59.50 ± 2.12	62.00 ± 1.41	60.50 ± 0.70	60.50 ± 0.70			
	28	61.00 ± 2.82	61.00 ± 2.82	61.50 ± 0.70	61.50 ± 2.12	60.00 ± 1.41			
FRAP assay (µmol Trolox/g sample)	1	33.64 ± 0.94^B^	40.07 ± 0.62^A^	40.29 ± 1.56^A^	37.63 ± 0.31^AB^	41.40 ± 1.25^A^	ND	**	ND
	7	34.09 ± 0.31^B^	37.85 ± 1.25^A^	40.73 ± 0.94^A^	37.19 ± 0.94^AB^	39.40 ± 0.94^A^			
	14	34.97 ± 0.94^B^	38.05 ± 1.88^AB^	41.18 ± 0.31^A^	37.85 ± 0.62^AB^	40.51 ± 1.88^A^			
	21	35.64 ± 1.25^B^	40.51 ± 2.50^AB^	41.62 ± 0.94^A^	38.07 ± 0.94^AB^	39.85 ± 0.94^AB^			
	28	35.19 ± 0.00^B^	39.18 ± 1.25^AB^	42.51 ± 3.44^A^	38.74 ± 0.00	38.30 ± 0.62^AB^			
L*	1	88.12 ± 4.16	92.19 ± 2.12	90.57 ± 3.01	91.88 ± 1.43^a^	90.67 ± 0.79	ND	*	ND
	7	89.72 ± 0.64	89.45 ± 0.62	86.55 ± 0.72	87.71 ± 2.58^ab^	87.49 ± 0.98			
	14	88.19 ± 0.18	86.06 ± 2.63	86.85 ± 0.51	87.67 ± 0.44^ab^	86.82 ± 0.82			
	21	89.15 ± 1.76	88.56 ± 1.80	86.88 ± 2.66	88.83 ± 1.01^ab^	88.10 ± 1.01			
	28	87.51 ± 0.62	89.27 ± 2.16	89.08 ± 0.02	85.93 ± 0.73^b^	88.55 ± 1.66			
a*	1 7 14 21 28	-3.44 ± 0.66	-3.71 ± 0.31	-3.20 ± 0.16	-3.54 ± 0.28	-3.26 ± 0.02	ND	*	ND
		-4.78 ± 0.18	-4.40 ± 0.04	-3.65 ± 0.64	-4.61 ± 0.50	-3.88 ± 0.60			
		-4.04 ± 0.38	-3.05 ± 0.17	-3.85 ± 0.47	-4.15 ± 0.57	-4.34 ± 0.01			
		-4.17 ± 0.98	-4.13 ± 0.67	-3.85 ± 0.28	-4.09 ± 0.03	-4.04 ± 0.23			
		-4.56 ± 0.65	-4.54 ± 0.71	-3.90 ± 0.58	-4.54 ± 0.59	-4.13 ± 0.09			
b*	1	5.84 ± 0.01^bB^	4.82 ± 0.01^bB^	8.97 ± 0.63^A^	6.20 ± 0.00^B^	7.81 ± 0.45^A^	**	*	ND
	7	6.12 ± 0.12^a^	6.29 ± 0.04^ab^	7.14 ± 0.41	5.67 ± 0.65	6.63 ± 1.16			
	14	5.26 ± 0.00^dB^	5.29 ± 0.01^abB^	7.56 ± 0.78^A^	4.56 ± 3.38^B^	7.31 ± 0.01^A^			
	21	5.84 ± 0.01^bAB^	5.33 ± 0.57^abB^	8.35 ± 1.27^A^	6.27 ± 0.70^AB^	7.62 ± 0.34^AB^			
	28	5.50 ± 0.01^cC^	6.70 ± 0.86^aB^	9.03 ± 0.65^A^	5.89 ± 1.65^C^	7.32 ± 0.70^B^			

*C* Goat milk + kefir culture (eXact Kefir-2), *LH100A *100% Lactose hydrolyzed goat milk + kefir culture (eXact Kefir-2), *LH100B* 100% Lactose hydrolyzed goat milk + kefir culture (XPL-1) + *S. boulardii*, *LH50A* 50% Lactose hydrolyzed goat milk + kefir culture (eXact Kefir-2) and *LH50B *50% Lactose hydrolyzed goat milk + kefir culture (XPL-1) + *S. boulardii*

a-d: Lower cases indicate that the Mean ± SD in the same column different from each other at *P* < 0.05.A-D: Upper cases indicate that the Mean ± SD in the same line different from each other at *P* < 0.05.*P < 0.05; **P < 0.001; NS, not significant *P* > 0.05, -type kefir sample, -type x -storage time: interaction between cheese sample and storage time

The total free amino acid content, reflecting this protein breakdown, increased significantly during kefir storage, from 0.28 to 0.37 Å on day 1 to 0.39–0.43 Å on day 28 (*P <* 0.05), indicating enhanced proteolytic activity. The changes in the absorbance values of the total free amino acid content in the kefir samples (Table 1). The progressive increase in free amino acids from day 1 to day 28 reflects sustained proteolytic activity during storage, independent of *S. boulardii* supplementation.These findings are agreement with the previous studies [41, 48].

Total free amino acid content in kefir samples made from lactose hydrolyzed samples (LH100A and LH50A) was significantly higher than in the control (*P <* 0.05) after 1st, 21st and 28th day of storage, while no significant difference was found between the free amino acid contents of the samples on the 7th and 14th days of storage (*P >* 0.05). Lactose hydrolysis can lead to an increase in proteolysis [50]; however, the similar values observed in samples LH100A and LH50A prepared from lactose-hydrolyzed milk without *S. boulardii* compared to samples LH100B and LH50B containing *S. boulardii* indicate that the addition of *S. boulardii* did not affect the extent of proteolysis.

### Antioxidant Activity

Antioxidants react differently to various radicals and oxidants, making it impossible to measure all components with a single method. Therefore, using multiple in vitro methods is beneficial [51]. In this study DPPH, ABTS, and FRAP assays were selected, which are commonly used in dairy products to measure both hydrophilic and lipophilic antioxidants [52]. For fermented dairy products, protein and peptide diversity, along with the microorganisms in the starter culture, are key factors influencing antioxidant activity [46]. The antioxidant activity results for kefir samples in this study showed variability due to the different mechanisms of the methods used [51].

ABTS values increased until day 14 and then declined towards day 28, indicating a transient accumulation of antioxidant peptides during mid-storage, followed by possible degradation in later stages, consistent with findings by Yılmaz-Ersan et al. [53]. A synergistic relationship between proteolysis products and phenolic compounds may enhance the total antioxidant potential of fermented products. The increase in antioxidant activity during storage can be attributed to lactic acid bacteria that reduce superoxide anions and hydroxyl radicals, and to the antioxidant activities of peptides formed during protein degradation [6, 54]. Peptides formed as a result of the fermentation of milk proteins also have antioxidant properties [46]. Amino acid levels are associated with antioxidant properties, and the decrease in activity can be attributed to the degradation of antioxidant peptides by proteolysis.

DPPH, a stable free radical, shows characteristic absorption at 515–520 nm. When converted to the non-radical form by hydrogen transfer, its color and absorbance decrease [46]. On day 1 of storage, DPPH values ranged from 60.5% to 61.5%, and on day 28, between 60.0% and 61.5%. No significant differences were found in DPPH inhibition activity (*P >* 0.05), indicating that neither lactose hydrolysis nor the addition of *S. boulardii* influenced the DPPH values. The high DPPH activity in the control sample is likely due to the superior antioxidant properties of goat milk [55, 56]. Gürel et al. [6] reported that goat milk kefir had higher antioxidant activity than other kefir samples.

FRAP, based on iron reduction capacity, is sensitive to pH changes, as iron solubility increases in acidic conditions [57]. In this study, no significant changes in pH occurred during storage, which may explain the stable FRAP activity (*P >* 0.05). Baniasadi et al. [46] noted that the increase in FRAP activity during goat milk kefir storage was linked to a decrease in pH (from 4.50 to 3.65).

### Carbohydrate Contents

The lactose content of the control sample made from non-hydrolyzed milk decreased from 3.27% on day 1 to 3.09% on day 28, with a decrease observed as storage progressed *P <* 0.05) as shown in Table 2. In kefir samples made from milk with 100% and 50% hydrolyzed lactose, the lactose content was between 0.08 and 0.13% and 1.75–1.97% respectively throughout the storage period. The decrease in lactose content is due to its hydrolysis to glucose and galactose, which is expected in kefir samples [58]. The lactose, glucose and galactose levels found in the control sample are similar to those reported by Bulat and Topcu [58]. In their study, Bulat and Topcu [58] reported that the lactose content in kefir samples decreased from 3.11 to 3.28% on the first day of storage to 2.94–3.14% on the 21st day, while the galactose content remained at the highest level of 0.02% and no glucose was detected in the samples.

**Table 2:** Carbohydrate and organic acid contents of kefir samples during storage

Variables	Kefir samples								ANNOVA		
	Storage (Day)	C (Control)		LH100A	LH100B	LH50A	LH50B		*P*-type	*P*-storage	*P*-type ⋅ storage
Lactose	1	3.27 ± 0.01^aA^		0.10 ± 0.01^E^	0.13 ± 0.00^D^	1.81 ± 0.00^aC^	1.97 ± 0.01^aB^		**	**	**
	7	3.19 ± 0.00^bA^		0.10 ± 0.00^D^	0.12 ± 0.00^D^	1.77 ± 0.00^bC^	1.91 ± 0.00^bB^				
	14	3.15 ± 0.01^cA^		0.10 ± 0.00^E^	0.12 ± 0.00^D^	1.78 ± 0.00^bC^	1.88 ± 0.00^bB^				
	21	3.12 ± 0.00^dA^		0.10 ± 0.00^E^	0.12 ± 0.00^D^	1.78 ± 0.00^bC^	1.89 ± 0.00a^bB^				
	28	3.09 ± 0.00^eA^		0.08 ± 0.00^E^	0.12 ± 0.00^D^	1.75 ± 0.00^bB^	1.84 ± 0.00^cC^				
Glucose	1	ND		2.42 ± 0.00^aA^	2.15 ± 0.00^B^	0.92 ± 0.00^aC^	0.37 ± 0.01^D^		**	**	**
	7	ND		2.30 ± 0.00^bA^	ND	0.74 ± 0.00^bB^	ND				
	14	ND		2.15 ± 0.00^cA^	ND	0.74 ± 0.00^bB^	ND				
	21	ND		2.02 ± 0.01^dA^	ND	0.63 ± 0.00^cB^	ND				
	28	ND		1.67 ± 0.00^dA^	ND	0.50 ± 0.00^dB^	ND				
Galactose	1	0.08 ± 0.01^cE^		4.47 ± 0.00^aB^	4.51 ± 0.00^aA^	2.13 ± 0.00^cD^	2.16 ± 0.00^aC^		**	**	**
	7	0.13 ± 0.01^bE^		4.44 ± 0.00^bA^	4.21 ± 0.02^cB^	2.14 ± 0.00^bC^	2.09 ± 0.00^cD^				
	14	0.16 ± 0.00^aE^		4.41 ± 0.00^cA^	4.18 ± 0.01^dB^	2.18 ± 0.00^aC^	2.09 ± 0.01^cD^				
	21	0.16 ± 0.00^aD^		4.41 ± 0.01^cA^	4.18 ± 0.00^dB^	2.14 ± 0.00^bC^	2.13 ± 0.00^bC^				
	28	0.14 ± 0.00^bE^		3.92 ± 0.00^dB^	4.23 ± 0.00^bA^	2.08 ± 0.00^dC^	2.08 ± 0.00^cC^				
Lactic acid	1	1357.94 ± 23.79^cBC^		1335.08 ± 0.99^bC^	1385.56 ± 1.82^cA^	1370.89 ± 0.82^B^	1308.18 ± 3.77^cD^			**	**
	7	1484.68 ± 5.61^bA^		1306.14 ± 3.48^cD^	1368.46 ± 2.80^abC^	1494.39 ± 0.33^A^	1417.89 ± 0.33^bB^				
	14	1498.0 ± 1.77^aB^		1474.99 ± 2.13^aC^	1358.93 ± 1.25^bcE^	1511.11 ± 0.95^A^	1417.49 ± 0.67^bD^				
	21	1493.81 ± 3.61^aB^		1474.20 ± 1.51^aC^	1364.44 ± 0.59^bE^	1507.33 ± 0.98^A^	1433.77 ± 0.02^aD^				
	28	1475.98 ± 7.11^bA^		1359.59 ± 2.67^bC^	1372.73 ± 0.08^aC^	1485.48 ± 1.50^A^	1398.79 ± 2.32^bB^				
Malic acid	1	12.08 ± 0.15^cB^		4.35 ± 0.11^aC^	4.29 ± 0.45^aC^	17.71 ± 2.41^bA^	1.86 ± 0.03^aC^		**	**	*
	7	14.84 ± 0.11^B^		4.24 ± 0.08^bC^	4.11 ± 0.26^b^	26.18 ± 0.27^aA^	1.84 ± 0.04^aD^				
	14	16.64 ± 0.11^abB^		4.05 ± 0.01^cC^	3.99 ± 0.26^bcC^	26.23 ± 0.10^aA^	1.57 ± 0.06^cD^				
	21	17.88 ± 0.10^aA^		3.58 ± 0.29^dB^	3.70 ± 0.23^cB^	2.04 ± 0.16^Cc^	1.80 ± 0.11^bC^				
	28	17.57 ± 0.19^aA^		4.24 ± 0.04^bB^	3.56 ± 0.18^C^	2.52 ± 0.21^cD^	1.59 ± 0.13^cE^				
Citric acid	1	3.80 ± 0.10^bA^		1.62 ± 0.04^cB^	1.48 ± 0.01^aBC^	1.36 ± 0.01^aC^	0.00 ± 0.00		**	**	**
	7	4.07 ± 0.01^aA^		2.02 ± 0.04^aB^	0.90 ± 0.00^dD^	1.26 ± 0.10^aC^	0.00 ± 0.00				
	14	3.38 ± 0.06^cA^		1.97 ± 0.02^aB^	1.08 ± 0.01^cC^	1.02 ± 0.01^bC^	0.00 ± 0.00				
	21	2.76 ± 0.05^dA^		1.76 ± 0.06^bB^	1.08 ± 0.00^cC^	1.05 ± 0.04^bC^	0.98 ± 0.01^aC^				
	28	2.35 ± 0.05^dA^		1.76 ± 0.06^bB^	1.16 ± 0.02^bB^	0.90 ± 0.01^cBC^	0.68 ± 0.22^bC^				
Acetic acid	1	43.56 ± 3.39^b^		40.89 ± 0.19^b^	41.71 ± 0.35^c^	39.17 ± 0.29^b^	41.46 ± 2.37^c^		**	**	**
	7	46.21 ± 0.56^aC^		42.61 ± 0.06^aC^	64.67 ± 0.75^bA^	45.17 ± 0.83^aC^	58.60 ± 1.81^bB^				
	14	43.35 ± 0.21^bC^		43.25 ± 0.32^aC^	68.16 ± 0.27^abA^	41.44 ± 0.36^bD^	59.74 ± 0.56^bB^				
	21	35.04 ± 0.55^cC^		35.21 ± 1.16^cC^	70.05 ± 0.35^aA^	35.02 ± 0.74^cC^	67.53 ± 0.11^aB^				
	28	33.42 ± 4.14^cB^		34.38 ± 0.11^cB^	70.07 ± 0.33^aA^	32.61 ± 0.62^cB^	64.03 ± 1.04^aA^				

*C* Goat milk + kefir culture (eXact Kefir-2), *LH100A* 100% Lactose hydrolyzed goat milk + kefir culture (eXact Kefir-2), *LH100B* 100% Lactose hydrolyzed goat milk + kefir culture (XPL-1) + *S. boulardii*, *LH50A* 50% Lactose hydrolyzed goat milk + kefir culture (eXact Kefir-2) and *LH50B* 50% Lactose hydrolyzed goat milk + kefir culture (XPL-1) + *S. boulardii*

a-d: Lower cases indicate that the Mean ± SD in the same column different from each other at *P* < 0.05A-D: Upper cases indicate that the Mean ± SD in the same line different from each other at *P* < 0.05*P < 0.05; **P < 0.001; NS, not significant *P* > 0.05, -type kefir sample, -type x -storage time: interaction between cheese sample and storage time

Kefir samples of LH100A and LH100B had higher glucose and galactose contents than the control and kefir samples made from milk with the samples of LH50A and LH50B. Increasing lactose hydrolysis from 50% to 100% resulted in markedly higher initial glucose (2.42% vs. 0.92%) and galactose levels, confirming effective enzymatic conversion.The lower levels of galactose in the samples spiked with *S. boulardii* (LH100B and LH50B) can be explained by the presence of *S. boulardii*, which is galactose positive and cannot ferment lactose, resulting in lower levels of galactose in the environment. As a result, *S. boulardii* begins to act as fermentation progresses in fermented dairy products, breaking down lactose into glucose and galactose through the activity of lactic acid bacteria [15, 21–23]. In the control sample, which had the lowest galactose content, an increase in galactose was observed during storage.

The glucose levels of kefir samples produced from 100% to 50% hydrolyzed lactose milk decreased during storage from 2.42 to 0.92% at the beginning of storage to 1.67 and 0.50% on day 28. No glucose was detected in the control sample throughout the entire storage period, while in the samples spiked with *S. boulardii* (LH100B and LH50B) glucose was only present on the first day of storage. This can be explained by the better utilization of glucose as a substrate by lactic acid bacteria [59]. In addition, the presence of *S. boulardii* in the same samples and its activity in breaking down lactose into glucose and galactose as fermentation progresses may explain this result [15, 23]. After hydrolysis of lactose into glucose and galactose, lactic acid bacteria metabolize glucose predominantly via a homofermentative pathway. Glucose is then utilized by lactic acid bacteria and metabolized mainly into lactic acid via pyruvate by the enzyme lactate dehydrogenase [47]. In conclusion, the observed differences in the glucose and galactose content of the kefir samples are related to the diversity of the microbial flora [58].

### Organic Acids

Organic acids play an important role in the flavor characteristics of fermented dairy products and contribute to their sensory properties and consumer acceptability. In addition, organic acids are considered natural preservatives due to their ability to inhibit certain pathogenic bacteria [60]. In kefir, which has a complex microbiota, one group of microorganisms can produce an organic acid that could be used as a substrate by another microbial group. This can potentially affect the production of other metabolites, which in turn can affect the sensory properties of the final product [58].

Lactic acid was the predominant organic acid in the kefir samples, with concentrations ranging from 1308.18 to 1511.11 mg/100 g (Table 2). It showed a fluctuating trend during the storage period (*P <* 0.05). Similarly, Sarıca and Coşkun [48] reported that the variation of lactic acid content during storage in kefir produced from cow and goat milk was related to the amount of lactic acid produced by lactic acid bacteria and its utilization by some yeast species.

The citrate contained in milk is used as a substrate by lactic acid bacteria in the Krebs cycle, resulting in the production of pyruvic acid, CO_2_ and acetic acid, and is also metabolized into volatile compounds such as acetaldehyde and diacetyl [48, 58]. Citric acid concentration in kefir samples was found to fluctuate during storage (*P <* 0.05), with the highest citric acid concentration consistently found in the control sample throughout the storage period. In a similar study by Sarıca and Coşkun [48], kefir samples from goat’s milk showed that citric acid levels increased until day 14, then to 35 µg/g on day 21 (*P <* 0.05).

Regarding the effect of lactose hydrolysis, it was found that the citric acid concentration was higher in kefir produced from 100% hydrolyzed lactose milk (LH100A) than in kefir produced from 50% hydrolyzed lactose milk (LH50A). In addition, no citric acid was detected in the kefir sample produced from 50% hydrolyzed lactose milk with *S. boulardii* (LH50B) during the first 14 days of storage, and the citric acid concentration detected thereafter was lower than in the other samples.

The malic acid concentrations in the kefir samples ranged from 1.57 to 26.23 mg/100 g and showed both increasing and decreasing trends during storage (*P <* 0.05). The highest malic acid concentration was found on days 1, 7 and 14 in the LH50A sample, while the highest concentration was found on days 21 and 28 in the control sample. A regular increase in malic acid concentration was observed in the control sample during storage. The lowest concentration of malic acid was found in all storage days in kefir samples produced from 50% hydrolyzed lactose milk with *S. boulardii* (LH50B).

Acetic acid is produced in kefir via three pathways: (1) by heterofermentative lactic acid bacteria through heterolactic fermentation, (2) by yeasts through oxidative decarboxylation of pyruvic acid using the enzyme pyruvate dehydrogenase and (3) by acetic acid bacteria through the two-step fermentation of carbohydrates [61, 58]. In this study, acetic acid concentrations ranged from 32.61 to 70.07 mg/100 g. the levels of acetic acid in the control and in the kefir samples produced from 100% to 50% hydrolyzed lactose milk (LH100A and LH50B) increased from the first to the 7th day and decreased from the 14th day onwards (*P <* 0.05). Unlike the control and hydrolyzed-only samples, acetic acid in LH100B and LH50B increased continuously throughout storage, indicating sustained metabolic activity associated with *S. boulardii*. The increase in acetic acid levels could be related to the increased metabolic activity of the microorganisms during fermentation. However, as fermentation progresses, some microorganisms can convert acetic acid into other metabolic products, leading to a decrease in its concentration [62].

### Rheological Properties

The consistency index (K) represents the resistance to shear stress, while the flow behavior index (n) indicates how easily the liquid deforms under shear stress. The values for the consistency index (K) and the flow behavior index (n) of kefir samples during storage are listed in Table 3. When examining the consistency index values, it was found that the values on days 1 and 28 of storage were between 2.59 and 4.64 and 1.98–3.76 Pa.s, respectively. Overall, there was fluctuations in the consistency index values during the storage and significant differences in the consistency index were found both between samples and storage days (*P <* 0.05). On the 28th day of storage, the highest consistency index values were found in kefir samples produced from 100% to 50% hydrolyzed lactose milk. The higher consistency index values observed in lactose-hydrolyzed samples on day 28 indicate improved structural resistance, potentially linked to enhanced microbial activity and polysaccharide interactions. The flow behavior index (n) is used to determine the proximity of the flow type to Newtonian flow. In Newtonian fluids, n equals 1, while in non-Newtonian flow types, if *n* > 1 is considered dilatant flow, and when *n* < 1 it is considered pseudoplastic flow [63]. A value of n approaching zero means that the product is inherently elastic. The flow behavior index values of the kefir samples ranged from 0.22 to 0.37 during storage. Since *n* < 1, according to the Power law model, the kefir samples were found to exhibit pseudoplastic (non-Newtonian) flow behavior during storage. It was observed that for all kefir samples, the flow behavior index values increased until day 14, decreased until day 21 and remained unchanged on day 28. Statistically significant differences in the flow behavior index were found between the samples and storage days *(P <* 0.05), while there were no significant differences between kefir samples’ flow behavior index values on different storage days (*P >* 0.05).

**Table 3:** Rheological properties and water holding capacity (WHC) of kefir samples during storage

Variables	Kefir samples						ANNOVA		
	Storage (Day)	C (Control)	LH100A	LH100B	LH50A	LH50B	P-type	P-storage time	P-type x storage
K (Pa.sn)	1	2.59±0.64abB	2.84±0.50bAB	3.93±0.40abAB	4.64±0.53aA	3.91±2.27abAB	*	**	ND
	7	3.85±0.09abB	4.29±0.41abA	4.39±0.07aA	4.60±0.14aA	4.24±0.20aA			
	14	4.47±0.17aA	4.64±0.31aA	3.50±0.27abB	4.33±0.24aA	3.99±0.28abB			
	21	4.06±0.03abA	4.50±0.46aA	2.72±0.48bcB	4.00±0.96aA	4.23±0.60aA			
	28	2.40±0.09bB	3.71±0.07abA	1.98±0.07cC	3.76±0.38bA	2.86±0.06bB			
n	1	0.26±0.01c	0.24±0.02b	0.25±0.01b	0.24±0.01c	0.23±0.01ab	ND	**	ND
	7	0.29±0.01a	0.25±0.07ab	0.26±0.02b	0.23±0.06bc	0.22±0.06b			
	14	0.36±0.01a	0.31±0.01a	0.37±0.01a	0.32±0.01a	0.35±0.01a			
	21	0.29±0.04b	0.26±0.02ab	0.32±0.01a	0.27±0.02b	0.27±0.01ab			
	28	0.29±0.01b	0.28±0.02a	0.32±0.01a	0.28±0.01b	0.27±0.01ab			
WHC (%)	1	51.03±0.53	52.65±4.20	54.22±1.64	53.05±3.33	50.88±0.48ab	*	*	ND
	7	48.83±1.11	50.95±2.46	53.42±3.20	51.23±2.46	47.97±1.27a			
	14	53.85±2.51	54.46±2.27	56.18±0.21	56.34±1.30	55.19±0.72b			
	21	53.50±0.98B	53.70±0.07B	56.73±1.03A	50.65±2.61C	50.41±2.60abC			
	28	51.44±3.34	51.73±0.76	54.92±1.35	50.57±4.39	49.13±1.16a			

*C* Goat milk + kefir culture (eXact Kefir-2), *LH100A* 100% Lactose hydrolyzed goat milk + kefir culture (eXact Kefir-2), *LH100B* 100% Lactose hydrolyzed goat milk + kefir culture (XPL-1)+ S. boulardii, LH50A:50% Lactose hydrolyzed goat milk + kefir culture (eXact Kefir-2) and *LH50B* 50% Lactose hydrolyzed goat milk + kefir culture (XPL-1)+ S. boulardii

a-d: Lower cases indicate that the Mean±SD in the same column different from each other at P < 0.05A-D: Upper cases indicate that the Mean±SD in the same line different from each other at P < 0.05*P < 0.05; **P < 0.001; NS, not significant P > 0.05, -type kefir sample, -type x -storage time: interaction between cheese sample and storage time

### Water Holding Capacity

The WHC is an important quality parameter in fermented dairy products and is considered a key function of proteins [64]. The WHC of the kefir samples ranged between 47.97% and 56.73%, with the highest value for the WHC observed on the 7th day of storage (Table 3); however, no significant difference was observed during storage (*P >* 0.05). On day 21, a statistically significant difference was found between the samples (*P <* 0.05). In general, the kefir samples produced from lactose hydrolyzed milk had higher WHC values than the control sample, and the highest WHC was consistently observed in the kefir sample produced from 100% hydrolyzed lactose milk with *S. boulardii* (LH100B) on all storage days. Similarly, İbrahim [49] reported that fermented milk prepared from camel milk with 90% hydrolyzed lactose had higher WHC than the control sample and the samples prepared from 25% to 50% hydrolyzed lactose milk. The researchers attributed this result to the higher content of exopolysaccharides found in the 90% hydrolyzed milk sample. In the same study, it was found that increasing lactose hydrolysis led to greater exopolysaccharide production during fermentation. However, no difference was observed in the water binding capacity of the samples with the addition of *S. boulardii* alone.

### Color

The changes in the L*, a* and b* color values of the kefir samples during storage are shown in Table 1. The L* values for the kefir samples ranged from 83.06 to 92.12 and the a* values from − 3.20 to -4.78 (Table 1). The b* values, indicating yellow color, ranged from 4.56 to 9.03 during storage (*P >* 0.05). It was found that kefir samples prepared from 100% to 50% hydrolyzed lactose milk with the addition of *S. boulardii* (LH100B and LH50B) had higher b* values than other kefir samples (*P <* 0.05).

### Volatile Compounds

Volatiles are natural organic compounds that are produced as secondary metabolites during microbial fermentation. The taste and aroma of kefir are due to the metabolic activity of the bacterial and yeast species present in the grain or starter culture used for production [65]. Acids, ketones, alcohols, aldehydes, esters and others are the predominant groups of volatile compounds in milk kefir [66]. In the study, the volatiles found in kefir were categorized into different chemical groups, including carboxylic acids (6), alcohols (11), aldehydes (7), esters (11), hydrocarbons (9), ketones (9) and sulfur (4) compounds (see Supplementary Table 1).

**Fig. 2:**
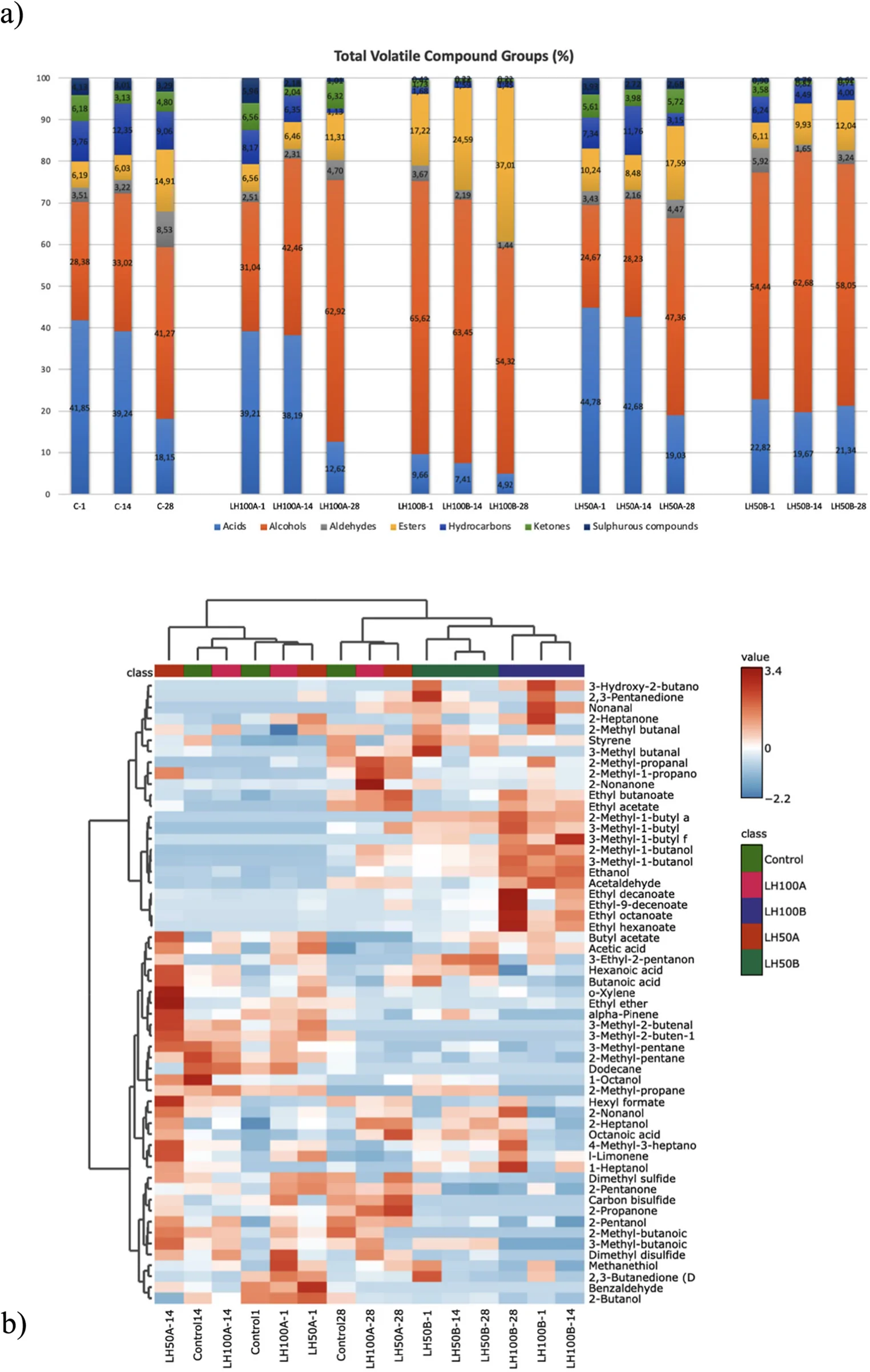
(**a**) Distribution (%) of volatile compound groups and (**b**) heatmap of volatile compound groups of kefir samples during 1, 14 and 28 day of storage. Control: Goat milk + kefir culture (eXact Kefir-2), LH100A:100% Lactose hydrolyzed goat milk + kefir culture (eXact Kefir-2), LH100B: 100% Lactose hydrolyzed goat milk + kefir culture (XPL-1) + *S. boulardii*, LH50A: 50% Lactose hydrolyzed goat milk + kefir culture (eXact Kefir-2) and LH50B: 50% Lactose hydrolyzed goat milk + kefir culture (XPL-1) + *S. boulardii*

When the volatile groups are examined, it is observed that the characteristic volatile compounds of the control sample and the LH50A and LH100A samples, which were produced from hydrolyzed milk, are acids (Fig. 2a). However, the characteristic volatile compounds of the kefir samples (LH50B and LH100B) produced from hydrolyzed milk with the addition of *S. boulardii* were identified as alcohols. This shift from acid-dominant to alcohol-dominant profiles in *S. boulardii*-containing samples clearly demonstrates its impact on fermentation pathways and aroma development. In the control, LH50A and LH100A samples, acids were found to decrease by the end of storage, with alcohols becoming the dominant group (Fig. 2a). Therefore, the dominant alcohol profile observed in the LH50B and LH100B samples was reached only at the end of storage in the other samples. These findings indicate that *S. boulardii* modifies fermentation pathways, leading to earlier dominance of alcohol-related aroma compounds.

#### Carboxylic Acids

Six carboxylic acids (acetic, butanoic, hexanoic, octanoic, 3-methyl-1-butanoic and 2-methyl-1-butanoic acids) were detected in kefir samples (see Supplementary Table 1). Acetic acid was the main acid in all kefir samples and the lowest level of acetic acid was detected in the control sample, which was produced from milk without added ß-galactosidase. LH50A and LH50B showed a higher concentration of butyric acid than all other kefir samples. Hexanoic acid (caproic acid) and octanoic acid (caprylic acid) were observed in all kefir samples at the beginning of storage and showed a tendency to first increase and then decrease during storage. The highest amounts of these acids were found in kefir samples LH50A and LH50B, which were produced from milk with 50% lactose hydrolysis without *S. boulardii* and with *S. boulardii*, respectively. The increase and decrease in the content of acids mainly produced by lipolysis can be related to the differences in the microbial population [67].

3-Methyl-1-butanoic and 2-methyl-1-butanoic acids were detected at low levels in kefir samples during the storage period, but not in the samples containing *S. boulardii* (LH100B). Also in kefir sample LH50B, no 2-methyl-1-butanoic acid was detected during the storage period. It is reported that the production of 3-methyl-1-butanoic acid is directly related to the esterification activities of yeast and bacterial species during fermentation and that the presence of certain microorganisms determines the level of this acid [68]. It is also known that the degradation of amino acids during the fermentation process plays an important role in the production of 2-methyl-1-butanoic acid and that the amount may decrease in the later stages of fermentation [69].

#### Alcohols

Among the alcohols, ethanol was the most important compound present in all kefir samples and it increased during storage due to lactose hydrolysis and the addition of *S. boulardii* by alcoholic fermentation. It is known that ethanol in kefir is mainly formed as a result of lactose fermentation and alcoholic fermentation of yeasts and its amount increases during storage [68]. While ethanol contributes to the overall flavour aroma, its presence in high amounts is undesirable, and high ethanol content can be associated with yeast aroma in kefir [70]. While the lowest ethanol content was determined in the control sample on all storage days, the highest amount of ethanol was determined in samples LH100B and LH50B, which were produced from lactose-hydrolyzed milk with the addition of *S. boulardii*.

3-Methyl-1-butanol and 2-methyl-1-butanol were detected in all kefir samples and showed an increasing trend during storage. However, the highest amounts were detected in kefir samples with *S. boulardii*. It is reported that 3-methyl-1-butanol is an important component of kefir and the increase in its amount can be associated with proteolytic activity [65]. It was found that the level of 2-heptanol increased during storage andthe lowest amount of 2-heptanol was detected in the control sample on all storage days. Other alcohols (2-butanol, 2-pentanol, 1-heptanol, 1-octanol and 2-nonanol) were determined in all kefir samples in similar concentrations and at a low level.

#### Aldehydes

Aldehydes are formed by transamination and decarboxylation of amino acids, Strecker degradation or lipid metabolism by microorganisms [65]. In this study, seven aldehyde compounds (acetaldehyde, 2-methyl-1-propanal, 3-methyl-1-butanal, 2-methyl-1-butanal, 3-methyl-3-butanal, benzaldehyde) were detected in kefir samples (see Supplementary Table 1). Acetic acid was the main acid in all kefir samples. The concentrations of these components in the different samples varied during the storage period. Of the aldehyde compounds, 2-methyl-1-propanal was detected during storage in LH100B and LH50B samples containing *S. boulardii*, while 3-methyl-2-butanal and benzaldehyde were not detected in the same samples. 2-Methyl-1-butanal was found in all kefir samples and showed an increasing trend during storage only in the control sample.

Acetaldehyde, an important volatile component in fermented dairy products [65], was the main aldehyde compound contributing to the flavor of the kefir samples. The study found that the amounts of acetaldehyde determined in the control, LH100A and LH50A samples on the first day of storage at 0.9, 1.91 and 1.33 mg/L, respectively, increased during storage and reached 1.33 mg/L on the 28th day of storage. The increase in the amount of acetaldehyde may be related to the fact that the specific enzyme activities that form acetaldehyde are greater than the ability of alcohol dehydrogenase to convert it to ethanol [71]. However, it was found that the amount of acetaldehyde determined at the beginning of storage in LH100B and LH50B samples with *S. boulardii* decreased during the storage. Similarly, Sarıca and Coşkun [48] found that the amount of acetaldehyde in kefir samples produced from goat and cow milk decreased during storage. The decrease in the amount of acetaldehyde is explained by the conversion of acetaldehyde to ethanol by the enzyme alcohol dehydrogenase. Nevertheless, the highest amount of acetaldehyde was tested in the LH100B sample produced from 100% lactose-hydrolyzed milk containing *S. boulardii* on all storage days, while the LH50B sample produced from 50% lactose-hydrolyzed milk containing *S. boulardii* had the second highest amount of acetaldehyde. *Saccharomyces* and other fermentative yeasts convert acetaldehyde into ethanol using the enzyme alcohol dehydrogenase, resulting in a high alcohol content [65]. The high ethanol content found in the samples in question confirms this result.

#### Esters

The esters found in kefir can be produced as secondary products of carbohydrate metabolism by both LAB and yeasts during fermentation. While at low concentrations they can make a positive contribution with a pleasant fruity aroma, at concentrations above 150 mg/L they can turn into a vinegar-like odor and cause an off-flavor in the product [65]. Among the 11 ester compounds, ethyl acetate and ethyl butonate were the most abundant compounds in all kefir samples. Their levels were found to increase during storage and were highest on day 28 in all samples: 1.15–6.97 and 12.92–28.36 mg/L, respectively (see Supplementary Table 1). Of the ester compounds, 3-methyl-1-butyl formate and 2-methyl-1-butyl acetate were detected during storage only in samples LH100B and LH50B with *S. boulardii*. It was also found that the highest amount of ethyl decanoate was found in LH100B and LH50B samples, and that ethyl decanoate increased during storage.

#### Hydrocarbons, Ketones and Sulfur Comounds

Hydrocarbons, which are secondary products of lipid oxidation, are also precursors for other aromatic compounds [65]. In the study, a total of 9 different hydrocarbon components (2-methyl-1-propane, ethyl ether, 2-methyl-pentane, 3-methyl-pentane, o-xylene, styrene, α-pinene, l-limonene, dodecane) were detected during the storage period, although their concentrations varied in the different samples. The most abundant hydrocarbon in high concentration among the nine was styrene.

Ketones are intermediates produced by the degradation of fatty acids or proteins in the fermentation process of microorganisms and can be reduced to alcohols. These compounds are obtained from pyruvate, a product of lactose and citrate metabolism. Many *Lactobacillus* species are able to metabolize citrate when another energy source such as lactose or glucose is present, resulting in the production of these volatile compounds [70]. Among the identified ketone compounds, 2-propanone was determined in high concentrations, but the compound was not identified in samples LH100B and LH50B containing *S. boulardii*. Diacetyl was found in all kefir samples. However, the highest levels were detected in samples LH100 and LH50. Similarly, Rutkowska et al. [70] found that diacetyl and acetoin dominated among the ketones identified in kefir from lactose-hydrolyzed milk and their amounts were about three and six times higher, respectively, than in control kefir. In our study, acetoin was only detected in sample LH100B, which was produced from lactose-hydrolyzed milk and contained *S. boulardii*, on all storage days.

The detected sulfur compounds generally showed a decreasing trend in the samples during the storage process. Methanethiol was the most important compound in all kefir samples, quantified in a range of 1.35 to 2.40 mg/L (see Supplementary Table 1). While the highest amount of dimethyl sulfide in all storage days was determined in the LH50B sample, the lowest dimethyl sulfide levels were determined in samples from lactose-hydrolyzed milk with added *S. boulardii*.

#### Heatmap of Volatile Compounds

The graph generated from the heatmap and cluster analysis of kefir samples is presented in Fig. 2. The samples are clustered into two distinct groups. The control, LH50A and LH100A samples, produced from lactose-hydrolyzed milk on days 1 and 14, are grouped together, while the LH50B and LH100B samples and all day 28 samples from the other group. This separation is consistent with the total acid and alcohol contents in the samples, as shown in Fig. 2. Groups with high acid content and those with high alcohol content are also separated into two groups. The control and LH50A and LH100A samples from days 1 and 14, combined on the left side of the graph, formed two separate clusters within themselves. This indicates that storage has a significant effect on the volatile compound composition of the samples. Additionally, the LH50B and LH100B samples, produced from lactose-hydrolyzed milk and containing *S. boulardii*, and the 28th day samples of the other groups, which merged on the left side of the graph, separated among themselves and formed two distinct groups. These results suggest that the use of *S. boulardii* has a significant effect on the volatile compound composition of kefir samples.

### Microbial Counts

**Fig. 3:**
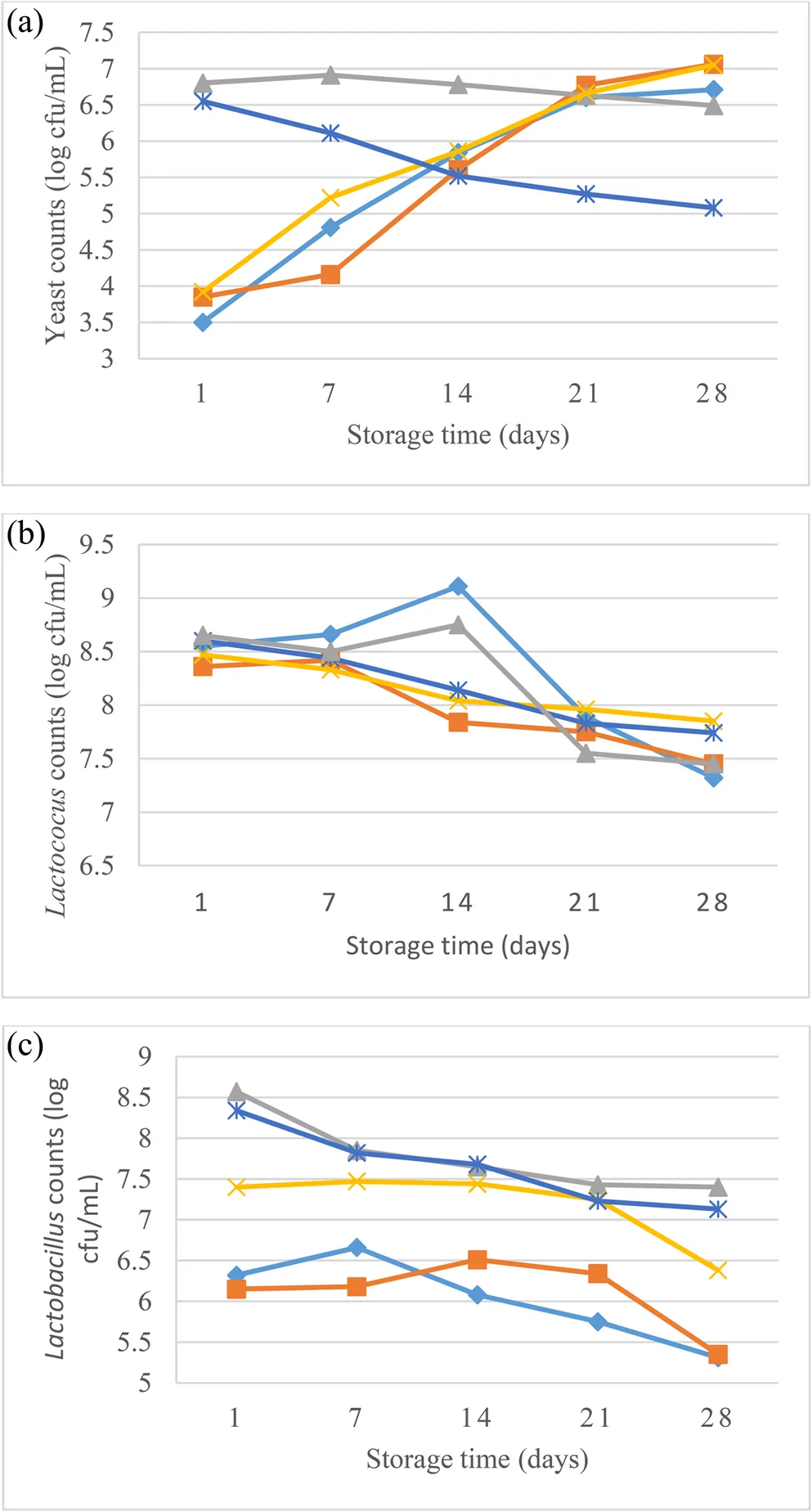
Growth of yeast (**a**), lactococci (**b**) and lactobacilli (**c**) during storage of kefir samples during storage at 4 °C. (υ) Control C: Goat milk + kefir culture (eXact Kefir-2), (ν) LH100A: 100% Lactose hydrolyzed goat milk + kefir culture (eXact Kefir-2), (π) LH100B: 100% Lactose hydrolyzed goat milk + kefir culture (XPL-1) + *S. boulardii*, (x) LH50A: 50% Lactose hydrolyzed goat milk + kefir culture (eXact Kefir-2) and (*) LH50B: 50% Lactose hydrolyzed goat milk + kefir culture (XPL-1) + *S. boulardii*

The changes in the number of *Lactococcus* spp., *Lactobacillus* spp. and yeast during the storage period of the kefir samples are shown in Fig. 3. The interaction between the treatments and the storage days was found to be significant for *Lactococcus* spp. and *Lactobacillus* spp. and yeast counts (*P <* 0.05). At the beginning of the storage period, the number of *Lactococcus* spp. was between 8.36 and 8.65 log CFU/mL. On day 28, these counts decreased to 7.36 to 7.85 log CFU/mL. The change in the number of *Lactococcus* spp. during storage was statistically significant (*P <* 0.05). The highest number of *Lactococcus* spp. was observed on day 14 in the control sample (*P <* 0.05). However, there were no significant differences in the *Lactococcus* spp. counts at the 1st, 7th, 21st, and 28th days (*P >* 0.05).

The number of *Lactobacillus* spp. in the kefir samples ranged from 6.15 to 8.57 log CFU/mL at the beginning of storage, and on day 28 this number decreased to 5.31 to 7.40 log CFU/mL (*P <* 0.05). The highest *Lactobacillus* spp. counts during the storage period were observed in the kefir samples produced from hydrolyzed milk with the addition of *S. boulardii* (LH100B and LH50B). The statistical differences in the number of *Lactobacillus* spp. between the samples were significant (*P <* 0.05). This result can be explained by the stimulating effect of *S. boulardii* on the viability of lactic acid bacteria, as reported in previous studies [21].

An increase in yeast count was observed during storage in the control (C), LH100A and LH50B samples (*P <* 0.05). However, a decrease was observed in the kefir samples with the addition of *S. boulardii* (LH100B and LH50B) produced from 100% to 50% hydrolyzed lactose milk (*P <* 0.05). The highest yeast counts during storage were observed in sample LH100B, made from 100% hydrolyzed lactose milk, on the 1st, 7th and 14th day of storage. The probiotic yeast *S. boulardii* maintained a viable count of at least 10^6^ CFU/mL throughout the storage period, which is generally considered to be beneficial to health. This stability confirms that lactose hydrolysis did not negatively affect probiotic survival capacity.

An important finding of this study is that the probiotic yeast *S. boulardii*, which cannot hydrolyze lactose, plays an essential role in maintaining the viability of lactic acid bacteria and yeast. Furthermore, yeast counts in the control (C) and in the samples produced from 100% to 50% lactose hydrolyzed milk (LH100A and LH50A) increased after day 21 and reached values of > 10^6^ CFU/mL.

### Sensory Evaluation

**Fig. 4:**
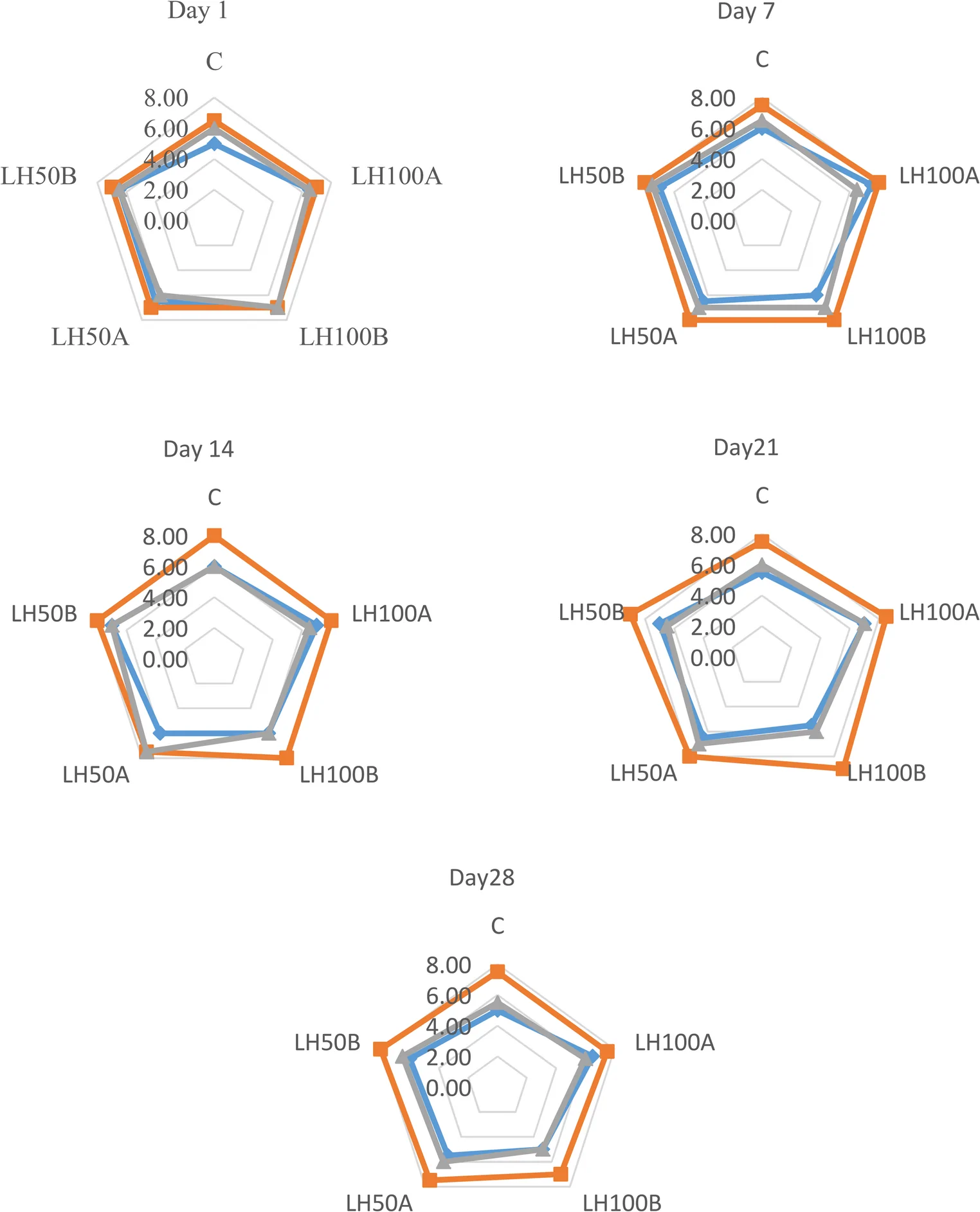
Sensory properties of kefir samples during storage at 4 °C. Control C: Goat milk + kefir culture (eXact Kefir-2), LH100A:100% Lactose hydrolyzed goat milk + kefir culture (eXact Kefir-2), LH100B: 100% Lactose hydrolyzed goat milk + kefir culture (XPL-1) + *S. boulardii*, LH50A: 50% Lactose hydrolyzed goat milk + kefir culture (eXact Kefir-2) and LH50B: 50% Lactose hydrolyzed goat milk + kefir culture (XPL-1) + *S. boulardii*. υ:Flavor, ν:Consistency, π:General acceptability

The changes in the taste-aroma, consistency and overall acceptability of the kefir samples during the 28-day storage period are shown in Fig. 4. Kefir sample made from 100% hydrolyzed lactose milk (LH100A) received the highest scores in terms of lactose hydrolysis. The sample made from 50% hydrolyzed lactose milk with *S. boulardii* (LH50B) received the highest score from the panelists and was thus the most preferred.

In terms of consistency, the control kefir sample was rated lower by the panelists during the 28-day storage period. However, both lactose hydrolysis and the addition of *S. boulardii* contributed to higher consistency scores, suggesting that these factors improved the panelists’ perception of the samples’ consistency.

When evaluating the overall acceptability of the samples, it was found that the addition of *S. boulardii* contributed positively to the overall acceptability of the kefir samples. In contrast, lactose hydrolysis alone did not cause a significant difference in overall acceptability. However, the kefir sample produced from 50% hydrolyzed lactose milk with *S. boulardii* (LH50B) was perceived as more acceptable by the panelists. Similarly, Karaolis et al. [14] reported that yogurt produced from goat’s milk with a mixture of lactic acid bacteria and *S. boulardii* had high sensory acceptability according to the panelists. These results suggest that the addition of *S. boulardii* plays an important role in enhancing the sensory properties of kefir, particularly in terms of taste-aroma, consistency and overall acceptability.This suggests that partial lactose hydrolysis may provide a more balanced sensory profile compared to complete hydrolysis, possibly due to moderated acid and alcohol formation.

## Conclusions

The findings of this study demonstrate that both lactose hydrolysis level and the incorporation of *S. boulardii* significantly influenced the physicochemical, rheological, microbiological, and sensory properties of goat milk kefir during storage. Partial lactose hydrolysis improved water-holding capacity, while the addition of *S. boulardii* enhanced microbial viability and contributed to favorable modifications in fermentation dynamics. Sensory evaluation indicated that kefir samples containing *S. boulardii* achieved higher overall acceptability scores, suggesting a positive contribution of probiotic yeast incorporation to consumer perception.

Among the tested formulations, LH50B (50% lactose hydrolysis combined with *S. boulardii*) emerged as the most promising sample, exhibiting a water-holding capacity of approximately 56.7%, maintaining yeast viability above 10⁶ CFU/mL throughout storage, and receiving the highest sensory acceptance. These results indicate that partial lactose hydrolysis, rather than complete hydrolysis, may provide a more balanced substrate environment that supports microbial metabolism while promoting desirable textural and flavour attributes.

Despite these promising outcomes, several limitations should be acknowledged. The study was conducted using a single goat milk matrix, a single β-galactosidase source, and one defined *S. boulardii* strain, which may limit the generalizability of the findings to other milk types, enzyme systems, or probiotic strains. Additionally, the experimental design was based on laboratory-scale production, and therefore does not fully reflect industrial processing variability. Moreover, probiotic functionality was inferred from viability data, without in vitro gastrointestinal simulation or clinical validation.

Future research should therefore expand this approach to different dairy matrices and alternative substrates, including plant-based systems, and evaluate probiotic persistence under simulated gastrointestinal conditions. Industrial-scale validation and stability studies, including potential microencapsulation strategies, would further support commercial applicability and functional product development.

## Supplementary Information

Below is the link to the electronic supplementary material.

Supplementary Material 1

## Data Availability

Even though adequate data has been given in the form of tables and figures, however, all authors declare that if more data is required then the data will be provided on a request basis.
