# Supplementary material for: An Innovative Kefir Produced from Lactose Hydrolyzed Goat Milk with the Addition of the Probiotic Saccharomyces Boulardii: Volatile Compound Profile, Rheological and Sensory Properties
Source: Probiotics Antimicrob Proteins. 2026 Mar 26;18(6):7898–916. doi: 10.1007/s12602-026-11002-0 (PMC13518513; doi:10.1007/s12602-026-11002-0)
Supplement: Supplementary file 1 [file 12602_2026_11002_MOESM1_ESM.docx]

**Supplementary Table 1.** Table of volatile compounds found in the samples 100% lactose hydrolyzed [without (LH100A) and with *S. boulardii* (LH100B)] and 50% lactose hydrolysed [without (LH50A) and with *S. boulardii* (LH50B)]

|  |  | **Samples** | | | | |
| --- | --- | --- | --- | --- | --- | --- |
| **Asitler** | **Days** | **C** | **LH100A** | **LH100B** | **LH50A** | **LH50B** |
| Acetic acid | **1** | 15.81±7.34 | 24.89±5.49 | 25.45±3,30 | 31.07±8.71 | 18.05±6.71 |
|  | **14** | 22.20±5.09 | 24.91±6.49 | 24.27±0.89 | 29.89±0.63 | 19.01±7.90 |
|  | **28** | 12.80±2.28 | 17.85±1.09 | 21.64±8.25 | 17.27±4.96 | 27.09±0.09 |
| Butanoic acid | **1** | 5.74±2.10 | 8.23±0.72 | 5.09±0.78 | 11.72±2.49 | 15.82±0.88 |
|  | **14** | 8.99±1.75 | 10.64±1.58 | 3.90±1.88 | 18.02±2.56 | 6.52±1.37 |
|  | **28** | 5.87±0.66 | 3.89±0.94 | 6.39±2.42 | 10.40±0.00 | 9.81±1.60 |
| 3-Methyl-butanoic acid | **1** | 0.16±0.05 | 0.45±0.04 | N.D. | 0.34±0.24 | 0.44±0.00 |
|  | **14** | 0.40±0.00 | 0.51±0.04 | N.D. | 0.81±0.18 | 0.44±0.00 |
|  | **28** | 0.45±0.15 | 0.65±0.00 | N.D. | 0.17±0.00 | 0.48±0.00 |
| 2-Methyl- butanoic acid | **1** | 0.07±0.00 | 0.33±0.06 | N.D. | 0.20±0.00 | N.D. |
|  | **14** | 0.31±0.00 | 0.28±0.05 | N.D. | 0.46±0.10 | N.D. |
|  | **28** | 0.51±0.00 | 0.27±0.00 | N.D. | N.D. | N.D. |
| Hexanoic acid | **1** | 7.89±3.26 | 14.11±1.11 | 14.89±4.54 | 20.03±4.73 | 21.97±6.02 |
|  | **14** | 18.70±8.10 | 21.36±2.58 | 12.17±0.00 | 37.42±2.72 | 23.44±0.64 |
|  | **28** | 10.03±0.96 | 11.85±2.79 | N.D. | 19.10±12.03 | 29.32±5.00 |
| Octanoic acit | **1** | 1.03±0.17 | 1.36±0.08 | 1.96±0.00 | 2.61±1.10 | 4.12±0.57 |
|  | **14** | 3.38±0.16 | 1.75±0.19 | 1.60±0.25 | 5.54±0.60 | 5.41±1.80 |
|  | **28** | 1.49±0.27 | 4.21±0.00 | 5.89±4.54 | 8.17±2.35 | 4.61±0.13 |
| **Alcohols** | **Day** | **C** | **LH100A** | **LH100B** | **LH50A** | **LH50B** |
| Ethanol | **1** | 12.84±3.78 | 25.27±2.87 | 209.38±60.29 | 21.00±1.17 | 90.35±7.62 |
|  | **14** | 31.20±9.70 | 51.53±5.18 | 242.93±15.27 | 31.09±3.78 | 113.55±37.65 |
|  | **28** | 32.53±12.51 | 92.96±15.72 | 237.63±3.73 | 63.38±31.24 | 121.87±40.51 |
| 2-Butanol | **1** | 1.51±0.33 | 1.58±0.19 | 0.37±0.13 | 1.81±0.17 | 0.39±0.00 |
|  | **14** | 1.02±0.00 | 0.70±0.00 | 0.15±0.00 | N.D. | 0.44±0.15 |
|  | **28** | 0.96±0.28 | 0.47±0.04 | 0.21±0.01 | 0.44±0.01 | 0.51±0.03 |
| 2-Methyl-1-propanol | **1** | 0.59±0.07 | 0.81±0.37 | 6.26±1.60 | 0.75±0.04 | 2.86±0.93 |
|  | **14** | 1.05±0.76 | 1.27±0.00 | 3.59±0.16 | 12.00±0.00 | 3.46±0.48 |
|  | **28** | 5.64±0.59 | 16.78±1.68 | 3.66±0.36 | 11.31±3.19 | 4.15±0.10 |
| 2-Pentanol | **1** | 0.95±0.56 | 2.10±0.32 | 1.66±0.15 | 1.71±0.90 | 1.46±0.26 |
|  | **14** | 1.62±0.70 | 2.31±0.76 | 0.74±0.13 | 2.54±0.67 | 1.42±0.16 |
|  | **28** | 2.69±0.56 | 2.27±0.00 | 1.00±0.08 | 2.36±0.95 | 1.67±0.13 |
| 3-Methyl-1-butanol | **1** | 0.71±0.39 | 1.00±0.17 | 72.63±5.67 | 1.49±0.23 | 32.53±1.70 |
|  | **14** | 2.00±0.00 | 0.90±0.09 | 87.79±5.34 | 0.97±0.13 | 35.81±9.30 |
|  | **28** | 15.65±6.66 | 52.22±12.61 | 98.14±3.29 | 35.55±13.33 | 42.12±12.79 |
| 2- Methyl-1-butanol | **1** | 0.35±0.00 | 0.41±0.00 | 25.20±10.70 | 0.64±0.28 | 9.01±0.66 |
|  | **14** | 0.44±0.38 | 0.33±0.06 | 18.78±0.19 | 0.33±0.20 | 10.07±0.57 |
|  | **28** | 6.13±1.08 | 16.44±3.65 | 23.19±0.35 | 12.09±4.25 | 12.17±0.69 |
| 3- Methyl-2-butan-1-ol | **1** | 0.26±0.13 | 0.37±0.04 | N.D. | 0.47±0.04 | N.D. |
|  | **14** | 0.34±0.00 | 0.34±0.00 | N.D. | 0.68±0.39 | N.D. |
|  | **28** | 0.28±0.08 | N.D. | N.D. | N.D. | N.D. |
| 2-Heptanol | **1** | 2.61±0.65 | 6.35±1.11 | 6.07±0.71 | 7.10±2.34 | 5.97±0.00 |
|  | **14** | 4.55±1.30 | 7.07±0.61 | 4.19±2.35 | 9.96±0.69 | 8.04±1.93 |
|  | **28** | 5.92±0.00 | 10.58±1.86 | 8.05±2.02 | 10.42±3.93 | 9.55±1.71 |
| 1-Heptanol | **1** | N.D. | N.D. | N.D. | N.D. | 0.39±0.00 |
|  | **14** | 0.30±0.13 | 0.30±0.00 | 0.46±0.28 | 0.59±0.21 | 0.23±0.00 |
|  | **28** | 0.19±0.04 | N.D. | 1.03±0.51 | N.D. | 0.43±0.00 |
| 1-Octanol | **1** | 0.25±0.03 | 0.18±0.03 | N.D. | 0.20±0.06 | 0.43±0.00 |
|  | **14** | 1.62±0.00 | 0.22±0.09 | N.D. | 0.90±0.15 | 0.27±0.00 |
|  | **28** | N.D. | N.D. | N.D. | 0.31±0.00 | 0.25±0.00 |
| 2-Nonanol | **1** | 0.74±0.21 | 1.01±0.06 | 0.52±0.00 | 0.20±0.06 | 0.72±0.00 |
|  | **14** | 1.29±0.16 | 1.10±0.07 | 0.70±0.01 | 0.90±0.15 | 1.37±0.02 |
|  | **28** | 0.89±0.08 | 1.25±0.93 | 2.02±0.53 | 0.31±0.00 | 1.33±0.11 |
| **Aldehydes** | **Days** | **C** | **LH100A** | **LH100B** | **LH50A** | **LH50B** |
| Acetaldehyde | **1** | 0.96±0.38 | 1.91±0.50 | 12.28±4.25 | 1.33±0.07 | 3.13±1.98 |
|  | **14** | 1.67±0.03 | 1.36±0.15 | 10.34±0.00 | 1.50±0.14 | 2.08±0.54 |
|  | **28** | 5.75±1.80 | 6.47±0.05 | 8.07±0.53 | 6.18±0.32 | 4.00±2.02 |
| 2-Methyl-propanal | **1** | N.D. | 0.72±0.00 | 2.69±1.02 | 0.36±0.00 | 0.89±0.22 |
|  | **14** | N.D. | N.D. | 0.74±0.21 | N.D. | 0.26±0.00 |
|  | **28** | 1.90±0.19 | 3.42±0.78 | 0.88±0.00 | 2.43±0.15 | 0.89±0.43 |
| 3-Methyl-butanal | **1** | N.D. | N.D. | N.D. | N.D. | 8.91±0.00 |
|  | **14** | 1.40±0.00 | 0.08±0.00 | N.D. | 0.95±0.00 | 0.17±0.00 |
|  | **28** | 4.98±1.51 | 2.39±0.00 | N.D. | 3.06±0.00 | 4.30±0.00 |
| 2-Methyl-butanal | **1** | 0.96±0.00 | N.D. | 2.42±0.97 | 2.18±0.25 | 2.40±0.00 |
|  | **14** | 1.15±0.00 | 2.09±0.00 | 0.94±0.40 | 1.77±0.88 | 1.84±0.00 |
|  | **28** | 1.89±0.08 | 1.90±0.87 | 0.96±0.10 | 0.95±0.00 | 1.44±0.00 |
| 3-Methyl-2-butanal | **1** | 0.02±0.00 | 0.05±0.01 | N.D. | 0.09±0.05 | N.D. |
|  | **14** | 0.05±0.00 | 0.07±0.00 | N.D. | 0.13±0.03 | N.D. |
|  | **28** | N.D. | N.D. | N.D. | N.D. | N.D. |
| Benzaldehyde | **1** | 0.64±0.21 | 0.48±0.18 | N.D. | 1.08±0.49 | N.D. |
|  | **14** | 0.16±0.03 | N.D. | N.D. | 0.31±0.00 | N.D. |
|  | **28** | 0.12±0.00 | N.D. | N.D. | N.D. | N.D. |
| Nonanal | **1** | N.D. | N.D. | 0.65±0.28 | N.D. | 0.34±0.16 |
|  | **14** | N.D. | N.D. | 0.41±0.00 | N.D. | 0.25±0.00 |
|  | **28** | N.D. | 0.23±0.02 | N.D. | 0.32±0.00 | 0.21±0.00 |
| **Esters** | **Days** | **C** | **LH100A** | **LH100B** | **LH50A** | **LH50B** |
| Ethyl acetate | **1** | 0.19±0.00 | 0.25±0.06 | 3.76±1.25 | 0.23±0.00 | 0.66±0.07 |
|  | **14** | 0.22±0.09 | 0.17±0.10 | 4.94±1.61 | 2.27±0.00 | 0.97±0.39 |
|  | **28** | 5.05±0.82 | 5.57±1.66 | 5.16±0.61 | 6.97±0.48 | 1.15±0.70 |
| 3-Methyl-1-butyl formate | **1** | N.D. | N.D. | 0.40±0.19 | N.D. | 0.37±0.22 |
|  | **14** | N.D. | N.D. | 1.29±1.01 | N.D. | 0.42±0.00 |
|  | **28** | N.D. | N.D. | 0.76±0.00 | N.D. | N.D. |
| Ethyl butanoate | **1** | 3.61±0.00 | 7.04±1.89 | 17.40±9.26 | 7.05±2.12 | 7.40±0.57 |
|  | **14** | 6.67±0.00 | 8.21±0.57 | 16.28±2.46 | 9.52±1.53 | 9.36±0.62 |
|  | **28** | 16.28±3.61 | 22.54±0.57 | 24.80±1.60 | 28.36±11.21 | 12.92±3.82 |
| Butyl acetate | **1** | 0.48±0.23 | 0.66±0.01 | 0.79±0.62 | 0.96±0.00 | 0.39±0.00 |
|  | **14** | 0.09±0.00 | 0.65±0.06 | 0.42±0.00 | 1.33±0.00 | 0.58±0.10 |
|  | **28** | N.D. | N.D. | 0.62±0.07 | N.D. | 0.71±0.33 |
| Hexyl formate | **1** | 0.12±0.08 | 0.20±0.02 | 0.16±0.00 | 0.25±0.00 | N.D. |
|  | **14** | 1.05±0.79 | 1.01±0.62 | 0.45±0.08 | 2.56±0.95 | 0.17±0.01 |
|  | **28** | 1.26±0.28 | 0.90±0.15 | 1.18±0.26 | 1.12±0.26 | 0.31±0.13 |
| 3-Methyl-1-butyl acetate | **1** | N.D. | N.D. | 0.69±0.21 | N.D. | 0.50±0.20 |
|  | **14** | N.D. | N.D. | 0.55±0.03 | N.D. | 0.52±0.02 |
|  | **28** | 0.34±0.00 | 0.21±0.02 | 1.10±0.11 | 0.74±0.00 | 0.56±0.07 |
| 2-Methyl-1-butyl acetate | **1** | N.D. | N.D. | 0.16±0.04 | N.D. | 0.13±0.00 |
|  | **14** | N.D. | N.D. | 0.15±0.00 | N.D. | 0.13±0.01 |
|  | **28** | N.D. | N.D. | 0.23±0.00 | N.D. | 0.14±0.02 |
| Ethyl hexanoate | **1** | N.D. | N.D. | 29.48±2.08 | 2.42±0.00 | 3.85±0.00 |
|  | **14** | N.D. | N.D. | 47.26±7.95 | N.D. | 6.83±2.17 |
|  | **28** | 1.88±0.86 | 4.00±0.57 | 96.64±14.57 | 9.12±1.41 | 12.86±0.00 |
| Ethyl octanoate | **1** | N.D. | N.D. | 30.12±0.00 | 3.24±1.65 | 2.32±0.44 |
|  | **14** | N.D. | N.D. | 61.74±16.54 | 2.14±0.66 | 7.29±2.66 |
|  | **28** | 0.54±0.07 | 1.28±0.31 | 109.55±53.57 | 2.97±0.94 | 9.77±2.37 |
| Ethyl-9-decenoat | **1** | N.D. | N.D. | N.D. | 0.14±0.04 | N.D. |
|  | **14** | N.D. | N.D. | 0.74±0.33 | N.D. | 0.22±0.00 |
|  | **28** | N.D. | N.D. | 2.01±1.16 | 0.44±0.00 | 0.26±0.00 |
| Ethyl decenoat | **1** | 0.14±0.00 | 0.12±0.02 | 1.60±0.30 | 0.80±0.32 | 0.55±0.08 |
|  | **14** | 0.27±0.05 | N.D. | 5.44±1.42 | 0.48±0.00 | 1.19±0.80 |
|  | **28** | 0.25±0.03 | 0.17±0.07 | 13.36±5.65 | 1.23±0.04 | 1.57±0.63 |
| **Hydrocarbons** | **Days** | **C** | **LH100A** | **LH100B** | **LH50A** | **LH50B** |
| 2-Methyl-propane | **1** | 1.06±0.62 | 1.12±0.27 | N.D. | 1.26±0.08 | 1.03±0.00 |
|  | **14** | 1.31±0.16 | 1.68±0.11 | N.D. | 1.10±0.51 | 1.19±0.17 |
|  | **28** | N.D. | N.D. | N.D. | N.D. | 1.15±0.00 |
| Ethyl ether | **1** | 1.87±0.77 | 3.13±0.92 | 0.74±0.00 | 2.50±0.09 | 0.45±0.41 |
|  | **14** | 0.10±0.00 | 0.19±0.13 | N.D. | 11.48±0.00 | 0.07±0.00 |
|  | **28** | 2.34±0.00 | N.D. | N.D. | N.D. | N.D. |
| 2-Methyl-pentane | **1** | 1.57±0.21 | 2.01±0.66 | 0.84±0.00 | 1.83±0.77 | 1.12±0.39 |
|  | **14** | 3.01±0.14 | 2.28±0.81 | 0.61±0.00 | 1.78±0.00 | 0.72±0.06 |
|  | **28** | 1.32±0.21 | 0.88±0.00 | 0.90±0.30 | 0.81±0.00 | 1.11±0.37 |
| 3-Methyl-pentane | **1** | 0.64±0.11 | 1.48±0.00 | 0.40±0.00 | 0.91±0.59 | 0.31±0.00 |
|  | **14** | 2.08±0.89 | 1.56±0.77 | 0.40±0.00 | 2.11±1.26 | 0.75±0.39 |
|  | **28** | 0.89±0.10 | 0.52±0.00 | 0.18±0.06 | 0.30±0.19 | 0.86±0.26 |
| o-Xylene | **1** | 0.55±0.13 | 0.71±0.23 | 0.51±0.15 | 1.42±0.56 | 0.64±0.42 |
|  | **14** | 0.81±0.09 | 0.80±0.43 | 0.42±0.13 | 2.51±0.00 | 0.56±0.20 |
|  | **28** | 0.69±0.17 | 0.54±0.01 | 0.83±0.16 | 0.70±0.34 | 0.67±0.15 |
| Styrene | **1** | 0.64±0.00 | 0.36±0.07 | 5.20±1.52 | 1.35±0.00 | 12.17±3.93 |
|  | **14** | 8.41±3.40 | 2.25±0.00 | 6.73±0.00 | 4.33±1.53 | 8.13±3.07 |
|  | **28** | 9.92±3.37 | 1.30±0.00 | 6.81±3.15 | 7.03±2.07 | 8.93±0.90 |
| 𝛼-Pinene | **1** | 0.22±0.09 | 0.20±0.19 | N.D. | 0.25±0.03 | 0.12±0.00 |
|  | **14** | 0.09±0.03 | 0.12±0.09 | N.D. | 0.42±0.13 | 0.22±0.00 |
|  | **28** | 0.10±0.00 | 0.07±0.01 | N.D. | N.D. | 0.10±0.00 |
| 1-Limonene | **1** | 0.36±0.00 | 0.89±0.00 | 0.54±0.24 | 1.30±0.15 | 0.68±0.32 |
|  | **14** | 0.71±0.35 | 0.57±0.39 | 0.77±0.42 | 1.67±0.00 | 0.86±0.63 |
|  | **28** | 0.21±0.01 | 0.15±0.02 | 1.26±0.08 | 0.29±0.00 | 0.57±0.12 |
| Dodecene | **1** | 0.25±0.17 | 0.39±0.03 | N.D. | N.D. | N.D. |
|  | **14** | 0.48±0.15 | 0.44±0.01 | N.D. | N.D. | N.D. |
|  | **28** | 0.09±0.00 | N.D. | N.D. | N.D. | N.D. |
| **Ketones** | **Days** | **C** | **LH100A** | **LH100B** | **LH50A** | **LH50B** |
| 2-Propanone | **1** | 1.96±0.49 | 3.51±1.07 | N.D. | 2.81±0.03 | 0.42±0.24 |
|  | **14** | 1.45±0.80 | 0.77±0.00 | N.D. | 4.82±0.00 | N.D. |
|  | **28** | 5.90±2.25 | 10.67±0.00 | N.D. | 13.17±0.00 | N.D. |
| Diacetyl | **1** | 0.83±0.39 | 1.41±0.65 | 0,85±0.54 | 1.17±0.27 | 1.67±0.70 |
|  | **14** | 0.26±0.08 | 0.10±0.02 | 0.03±0.00 | 0.18±0.13 | 0.14±0.00 |
|  | **28** | 0.22±0.15 | 0.35±0.18 | N.D. | 0.31±0.25 | 0.04±0.02 |
| 2-Pentanone | **1** | 0.47±0.12 | 1.03±0.75 | 0.70±0.39 | 1.12±0.05 | 0.80±0.24 |
|  | **14** | 0.72±0.45 | 0.76±0.25 | 0.24±0.00 | 0.61±0.11 | 0.23±0.00 |
|  | **28** | 0.94±0.67 | 0.80±0.00 | 0.28±0.00 | 1.00±0.92 | 0.18±0.10 |
| 2,3-Pentandion | **1** | N.D. | N.D. | 0.56±0.51 | 0.20±0.00 | 0.75±0.50 |
|  | **14** | N.D. | N.D. | N.D. | N.D. | 0.17±0.00 |
|  | **28** | N.D. | 0.09±0.00 | N.D. | 0.23±0.00 | N.D. |
| Acetoin | **1** | N.D. | N.D. | 2.54±0.25 | 0.19±0.27 | 2.01±0.00 |
|  | **14** | N.D. | N.D. | 1.33±0.00 | N.D. | N.D. |
|  | **28** | N.D. | N.D. | 1.04±0.08 | N.D. | 0.04±0.05 |
| 3-Ethyl-2-pentanone | **1** | 0.05±0.01 | 0.13±0.05 | 0.13±0.00 | 0.10±0.07 | 0.12±0.02 |
|  | **14** | N.D. | N.D. | N.D. | 0.12±0.05 | 0.21±0.00 |
|  | **28** | N.D. | N.D. | N.D. | N.D. | 0.22±0.00 |
| 2-Heptanone | **1** | 0.53±0.16 | 0.85±0.23 | 1.57±0.64 | 1.15±0.00 | 0.91±0.00 |
|  | **14** | 0.40±0.17 | 0.33±0.03 | 0.52±0.10 | 0.51±0.00 | 0.33±0.16 |
|  | **28** | 0.42±0.19 | 0.43±0.00 | 0.93±0.53 | 0.46±0.29 | 0.52±0.44 |
| 4-Methyl-3-heptanone | **1** | 0.48±0.00 | 1.23±0.19 | 0.79±0.26 | 1.30±0.32 | 1.23±0.81 |
|  | **14** | 1.19±0.52 | 1.21±0.01 | 0.65±0.37 | 2.34±0.76 | 0.99±0.20 |
|  | **28** | 0.69±0.25 | 0.53±0.03 | 2.23±0.50 | 0.96±0.24 | 1.18±0.46 |
| 2-Nonanon | **1** | 0.22±0.00 | 0.10±0.02 | 1.36±0.11 | 0.22±0.13 | 1.57±0.56 |
|  | **14** | 0.29±0.04 | N.D. | 0.31±0.00 | N.D. | 0.20±0.03 |
|  | **28** | 0.08±0.00 | 6.52±0.00 | N.D. | 0.44±0.00 | 0.18±0.00 |
| **Sulfur Compounds** | **Days** | **C** | **LH100A** | **LH100B** | **LH50A** | **LH50B** |
| Methanethiol | **1** | 1.35±0.52 | 2.40±0.50 | 1.87±0.00 | 1.81±0.18 | 2.05±0.07 |
|  | **14** | 1.57±0.30 | 1.69±0.29 | 1.19±0.00 | 1.53±0.42 | 1.43±0.32 |
|  | **28** | 1.45±0.37 | 1.62±0.08 | 1.28±0.12 | 1.83±0.28 | 1.42±0.21 |
| Dimethyl sulfide | **1** | 1.26±0.46 | 3.40±0.05 | 0.20±0.03 | 3.85±0.00 | 0.33±0.01 |
|  | **14** | 2.40±0.21 | 0.99±0.19 | 0.08±0.00 | 3.44±0.00 | 0.65±0.22 |
|  | **28** | 3.36±1.65 | 0.20±0.00 | 0.25±0.00 | 4.48±0.00 | 0.49±0.60 |
| Carbon bisulfide | **1** | 0.36±0.00 | 0.92±0.24 | N.D. | N.D. | N.D. |
|  | **14** | N.D. | 0.20±0.00 | N.D. | 0.46±0.00 | 0.02±0.01 |
|  | **28** | 0.77±0.00 | 0.69±0.00 | N.D. | 1.15±0.00 | 0.01±0.00 |
| Dimethyl disulfide | **1** | 0.06±0.01 | 0.79±0.00 | N.D. | 0.14±0.03 | N.D. |
|  | **14** | 0.17±0.00 | 0.52±0.00 | N.D. | 0.44±0.29 | 0.02±0.00 |
|  | **28** | 0.05±0.02 | 0.57±0.24 | N.D. | 0.32±0.02 | 0.15±0.17 |
